# CDCA5 accelerates progression of breast cancer by promoting the binding of E2F1 and FOXM1

**DOI:** 10.1186/s12967-024-05443-w

**Published:** 2024-07-08

**Authors:** Yiquan Xiong, Lan Shi, Lei Li, Wen Yang, Huiqiong Zhang, Xiangwang Zhao, Na Shen

**Affiliations:** Department of Breast and Thyroid Surgery, Union Hospital, Tongji Medical College, Huazhong University of Science and Technology, 1277 Jiefang Road, Wuhan, Hubei 430022 China

**Keywords:** CDCA5, Breast cancer, FOXM1, Wnt/β-catenin signaling

## Abstract

**Background:**

Breast cancer is one of the most common malignant tumors in women. Cell division cycle associated 5 (CDCA5), a master regulator of sister chromatid cohesion, was reported to be upregulated in several types of cancer. Here, the function and regulation mechanism of CDCA5 in breast cancer were explored.

**Methods:**

CDCA5 expression was identified through immunohistochemistry staining in breast cancer specimens. The correlation between CDCA5 expression with clinicopathological features and prognosis of breast cancer patients was analyzed using a tissue microarray. CDCA5 function in breast cancer was explored in CDCA5-overexpressed/knockdown cells and mice models. Co-IP, ChIP and dual-luciferase reporter assay assays were performed to clarify underlying molecular mechanisms.

**Results:**

We found that CDCA5 was expressed at a higher level in breast cancer tissues and cell lines, and overexpression of CDCA5 was significantly associated with poor prognosis of patients with breast cancer. Moreover, CDCA5 knockdown significantly suppressed the proliferation and migration, while promoted apoptosis in vitro. Mechanistically, we revealed that CDCA5 played an important role in promoting the binding of E2F transcription factor 1 (E2F1) to the forkhead box M1 (FOXM1) promoter. Furthermore, the data of in vitro and in vivo revealed that depletion of FOXM1 alleviated the effect of CDCA5 overexpression on breast cancer. Additionally, we revealed that the Wnt/β-catenin signaling pathway was required for CDCA5 induced progression of breast cancer.

**Conclusions:**

We suggested that CDCA5 promoted progression of breast cancer via CDCA5/FOXM1/Wnt axis, CDCA5 might serve as a novel therapeutic target for breast cancer treatment.

**Supplementary Information:**

The online version contains supplementary material available at 10.1186/s12967-024-05443-w.

## Background

Breast cancer is one of the most common malignant tumors in women [1]. The incidence of breast cancer accounts for more than 11.6% of female tumors, which is second only to uterine cancer in women [2]. Surgery, radiotherapy and chemotherapy are still the main therapy methods for breast cancer, while they are only suitable for patients in early stage, and have the limitations of easy recurrence and large side effects [3, 4]. Recently, results of phase III clinical trials have shown that immune checkpoint inhibitors, such as atezolizumab and pembrolizumab, are well-tolerated in combination with chemotherapy, benefit for progression-free survival of patients with triple-negative breast cancer (TNBC) [5, 6]. These findings suggest that immunotherapy emerges as a viable treatment strategy for breast cancer. However, immune checkpoint inhibitors alone exhibit modest clinical activity in advanced breast cancer, thus developing more active combinatorial modalities and more effective biomarkers are needed to increase survival rate of patients with breast cancer [7]. Therefore, there is a critical need to uncover novel and effective therapeutic targets to improve survival rate of breast cancer patients.

Cell division cycle associated 5 (CDCA5), also termed as sororin, is a master regulator of sister chromatid cohesion and separation [8]. CDCA5 plays pivotal role in stabilizing cohesion of chromatids during S and G2/M cell cycle phases, as well as maintaining and repairing stability of DNA strands during G2 phases [9–11]. In recent years, CDCA5 was reported to function as a tumor promoter in various tumors, including bladder cancer, hepatocellular carcinoma [12, 13], gastric cancer [14, 15] and esophageal squamous cell carcinoma [16], as well as the breast cancer [17–19]. Moreover, new data suggested that upregulation of CDCA5 is correlated with prognosis of cancers and may be an independent predictor of cancer outcome, including breast cancer [20, 21]. Although bioinformatics has initially identified the abnormal upregulation of CDCA5 in breast cancer, the specific function and mechanism had not yet been elucidated.

Herein, we showed the upregulation of CDCA5 in breast cancer, and we used shRNA-mediated knockdown of CDCA5 to explore functional role of CDCA5 in breast cancer. Mechanistically, we found that CDCA5 promoted the binding of E2F transcription factor 1 (E2F1) to FOXM1 promoter, and Wnt/β-catenin signaling was required for CDCA5 induced development of breast cancer. CDCA5 might become a potential therapeutic target for breast cancer treatment.

## Materials and methods

### Clinical tissue specimen analysis

A total of 96 breast cancer tissues and 22 para-carcinoma tissues were included in this tissue microarray for immunohistochemical (IHC) analysis. The tissue microarray (Cat No. HBreD136Su02) was purchased from Shanghai Outdo Biotech Company. The clinicopathologic information of patients and their written informed consent for this research were provided and this study was approved by the Ethics Committee of Union Hospital, Tongji Medical College, Huazhong University of Science and Technology (approval no. 2023 − 421). For IHC staining, the embedded tissues were subjected to dewax, rehydrate, antigen repair firstly. Afterwards, the primary antibody anti-CDCA5 and corresponding secondary antibody was added and incubated with tissue slides. The diaminobenzene (DAB) and hematoxylin were applied for staining. The IHC scores were determined by percentages of positive staining cells and the staining intensity. The percentages of positive staining cells and staining intensity were scored 1 ~ 4 and 0 ~ 3, respectively. For the former: 1, 0 ~ 24%; 2, 25 ~ 49%; 3, 50 ~ 74%; 4, 75 ~ 100%; for the latter, 0, no staining signals; 1, light yellow; 2, pale brown; 3, seal brown. Finally, the IHC scores were as follows: 0 score (-), 1–4 scores (+), 5–8 scores (++), 9–12 scores (+++) [22].

### Cell culture and treatment

The normal MCF-10 A, human breast cancer cell lines (BT-549, MDA-MB-231, T47D, MCF-7, MDA-MB-453 and MDA-MB-468) and the 293T cells were all purchased from the American Type Culture Collection (ATCC) (Manassas, USA). The BT-549 was cultured in RPMI-1640 medium (Gibco, USA), MCF-10 A, T47D, MDA-MB-468, MCF-7 and 293T were grown in DMEM-high glucose medium (Gibco, USA), MDA-MB-231 and MDA-MB-453 were maintained in L15 medium (Hyclone, USA), respectively. All culture medium were supplemented with 10% fetal bovine serum (FBS) (Gibco, USA) and 1% Penicillin/Streptomycin (100 U/mL). MDA-MB-231 cells were incubated at 37 °C without CO_2_, the other cells were maintained in a 5% CO_2_ incubator at 37 °C. Wnt/β-catenin inhibitor C59 (Cat No. HY-15,659, MCE, China) was used at 20 µmol/L for 24 h after lentivirus transfection.

### RNA interference and overexpression

RNA interference sequence for CDCA5 (shCDCA5), FOXM1 (shFOXM1), CDCA5 and E2F1 overexpressing sequences as well as corresponding scrambling sequence (shCtrl, negative control) were obtained from Shanghai YiBR Bioscires (Shanghai, China). The targeted sequences of shRNAs were shown in Table S1, the sequence of the shRNA used as control in this study was TTCTCCGAACGTGTCACGT. According to the manufacturer’s protocol, the RNA interference sequence was connected to lentivirus BR-V108 vector (YiBR, China) and co-transfected with pMD2.G (Qiagen, China) and pSPAX2 (Qiagen, China) using 293T cells to generate recombinant lentivirus plasmids (shCDCA5 and shFOXM1). Subsequently, BT-549 or MDA-MB-231 cells were plated at 2 × 10^6^ cells per well and transfected with 10 µg of the indicated plasmids. The stable cell lines expressing shCDCA5 or shFOXM1 were selected with puromycin as previously described [23]. Construction of recombinant lentivirus containing amplified sequence of CDCA5 (CDCA5 overexpression) or E2F1 (E2F1 overexpression) in subsequent experiment was accomplished using similar methods as above.

### Real-time quantitative PCR (qPCR)

The relative mRNA levels of targeted genes were determined according to manufacturer’s instructions. In brief, the total RNA was isolated from cellular specimens using TRIzol regent (Sigma, USA) followed by its quantification by Nanodrop 100 (Thermo, USA). Complementary DNA (cDNA) was synthesized using the Hiscript QRT supermix (Vazyme, China). The qPCR reaction (10 µL) was then performed by SYBR Green mastermixs Kit (Vazyme, China) and Biosystems 7500 Sequence Detection system. The thermocycling conditions used in RT-qPCR were as follows: initial denaturation at 95˚C for 30 s, followed by 40 cycles of 95˚C for 5 s, annealing at 60˚C for 30 s and followed by 1 cycle of 95˚C for 15 s, at 60˚C for 30 s and 95˚C for 15 s. Each reaction was performed in triplicate, and expression values were normalized to internal control GAPDH. Relative quantitative expression of genes was calculated by the 2^−ΔΔCt^ method. Primer sequences were given in Table S2.

### Western blotting (WB)

The total proteins of cellular samples were collected by radioimmunoprecipitation (RIPA) lysis followed by protein quantification using BCA Protein Assay Kit (HyClone-Pierce, USA). 20 µg protein lysates from each specimen were separated by 10% sodium dodecyl sulfate polyacrylamide gel electrophoresis (SDS-PAGE) (Invitrogen, USA). The proteins were then electroblotted on polyvinylidene difluoride (PVDF) membranes and blocked with 5% slim milk for 1 h. The blocked membranes were then incubated with primary antibodies overnight at 4 °C. Next, the secondary horseradish peroxidase (HRP)-conjugated antibody was added for 2 h incubation. The relative protein levels were visualized by enhanced chemiluminescence (ECL, Millipore). All antibodies used in this study were listed in Table S3. GAPDH was used as an internal reference.

### Celigo cell counting assay

Cells were firstly transfected with corresponding lentivirus. After 48 h transfection, cells were then harvested, and seeded into 96-well plates at a density of 2 × 10^3^ cells per well. From the second day after cell inoculation, the plate was read once a day for 5 consecutive days by the Celigo image cytometer (Nexcelom Bioscience, LLC). Since Celigo is a high-throughput screening system with fully automated image acquisition and image data analysis, the number of cells contained in each group in the well plate could be calculated by fluorescence excitation of the target cells expressing GFP (excitation wavelength was 488 nm, and the emission wavelength was 509 nm) after infection with the lentivirus. Finally, based on the cell count data of the Celigo instrument, we can then plot the cell growth curve, reflecting the proliferation status of the cells.

### Colony formation assay

After transfection of lentivirus to breast cancer cells for 48 h, cells were digested and re-suspended. The prepared cells were then plated into 6-well plates at a density of 500 cells per well and cultured for an additional 2 weeks. Finally, the cell colonies were stained with GIEMSA following 4% paraformaldehyde fixation and photographed.

### Apoptosis assay

Cells were seeded at a density of 1 × 10^6^ cells per well in 6-well plates and transfected with lentivirus when reaching 70% confluence for 24 h. Subsequently, cells were harvested, re-suspended in 200 µL of 1 × binding buffer, and stained with Annexin V-APC and PI-PE in the dark for 15 min. Apoptotic cells were then quantified using a flow cytometer (Millipore Guava easyCyte HT, Millipore Sigma) and analyzed with GuavaSoft 3.0 (Millipore Sigma, GER).

### Cell cycle assay

The breast cancer cells transfected with indicated lentivirus were re-suspended with 200 µL PBS buffer followed by fixing with 70% ethyl alcohol for 1 h. Then the cells were washed three times using PBS and stained with Propidium lodide (PI) solution (Sigma, USA) (40 × PI (2 mg/mL): 100 × RNase (10 mg/mL): 1 × PBS = 25: 10: 1000) for 10 min away from light. Finally, the cells distributed in different stage were quantified by flow cytometer (Millipore Guava easyCyte HT, Millipore Sigma) and analyzed by GuavaSoft 3.0 (Millipore Sigma, GER).

### Wound healing assay

The transfected breast cancer cells in logarithmic phase were trypsinized, re-suspended and counted. Then cells were seeded into 96-well plates at a density of 5 × 10^4^ cells per well. The next day when cells were at 90% confluence, the scratch was generated by a scratch tester from the bottom center of the 96-well plate. Cells were cultured in medium with 0.5% FBS following FBS-free medium washing. The wound healing was observed by Cellomics (Thermo, USA) at indicated time points, and the migration rate was calculated as scratch width difference (migratory distance of the cell at indicated time points (48 h/24 h)-0 h scratch width)/0 h scratch width.

### Transwell assay

According to the manufacturer’s instructions of transwell assay kit (Corning 3422 with 8 μm membrane pore size, USA), the transfected BT-549 and MDA-MB-231 cells were collected and re-suspended in FBS-free medium. 100 µL of cell suspensions were added into the upper chamber. Next, 600 µL of medium supplemented with 30% FBS was filled in the lower chambers. The upper chamber containing cells were transferred into the lower chamber. After 24 h of incubation at 37 °C with 5% CO2, cells on the upper surface of membrane were removed with cotton tip, and cells on the lower surface were stained with 0.1% crystal violet for 5 min following fixation of 4% paraformaldehyde. 5 fields of each well were selected and counted under 200 × microscope (IX73, Olympus, Japan). The migratory cell number was determined by average cell number of the 5 microscopic views.

### Affymetrix Human Gene Chip Prime View

Affymetrix human Gene Chip Prime View combined with Affymetrix Scanner 3000 was used to analyze the potential targets of CDCA5. The differentially expressed genes (DEGs) between shCDCA5-depleted and its empty control MDA-MB-231 cells, were screened by criterion of |Fold Change| ≥ 1.3 and false discovery rate (FDR) < 0.05. The DEGs were then presented as heat map of Hierarchical Clustering analysis. The potential downstream targets were analyzed by constructing interaction network depending on the Ingenuity Pathway Analysis (IPA).

### Co-immunoprecipitation (Co-IP)

The Co-IP experiments were performed as described previously [24]. Briefly, the MDA-MB-231 cells lysates were prepared in RIPA buffer and quantified by BCA kit (HyClone-Pierce, USA). 1.0 mg total proteins were incubated with anti-E2F1 at 4 °C overnight followed by incubation of 20 µL agarose beads at 4 °C for 2 h. Conjugation product of proteins-antibody-beads was then separated by 2000 g of centrifugation for 1 min and 5 min of lysis with IP lysate buffer at 100 °C. The immunoprecipitated proteins were subjected to WB analysis as described above. Antibodies used in Co-IP were listed in Table S3.

### Dual-luciferase reporter assay

The FOXM1 promoter region (− 2000, + 100) was amplified and the resulting fragment was cloned into the luciferase reporter vector GL002 (Promega Madison, USA), designated as GL002-FOXM1. Mutant construct GL002-FOXM1-Mut was generated by site-directed mutagenesis. Luciferase assay was performed as described previously [25]. Each experimental analysis was repeated three times.

### Chromatin immunoprecipitation (ChIP) assay

The ChIP assay was performed using the SimpleChIP^®^ Enzymatic Chromatin IP Kit (Cat No, 9002 S, CST, USA) according to the manufacturer’s instructions. In brief, the MDA-MB-231 cells were transfected with CDCA5-overexpressing vector or its empty vector and incubated for 48 h at 37 °C with 5% CO_2_. When cell grown to 90% confluence, the cells were crosslinked with 37% formaldehyde followed by lysed in SDS buffer and sheared sonication to fragment the DNA. Afterwards, the sonicated chromatin was precipitated by incubating it with according antibody overnight at 4 °C. The Protein–DNA complexes were then purified and the purified DNA was dissolved in nuclease-free water followed by qPCR analysis using the primers of FOXM1 promoter and SYBR Green I Master (Roche, USA). The primers used for FOXM1 promoter amplification were shown in Table S2. Antibodies used in this assay were listed in Table S3.

### Mice xenograft model

Sixteen female BALB/c nude mice (Four-weeks-old, 13 ± 1 g) were purchased from GemPharmatech Co., Ltd. (Jiangsu, China) and housed in the specific-pathogen-free (SPF) condition. The holding room was kept at a temperature range of 20 to 26 °C, with humidity between 30% and 70%, and followed a 12-hour light/dark cycle. The mice had unrestricted access to standard laboratory food and clean drinking water. The animal facility was adequately ventilated, and all cages were consistently maintained in a clean and sanitary condition to prioritize the well-being and health of the animals. For the construction of xenograft models, the CDCA5-overexpressed, FOXM1-depleted, CDCA5-overexpressed + FOXM1-depleted and control MDA-MB-231 cells were prepared and suspended in 200 µL of PBS (1 × 10^7^ cells/mL). Then the cells were subcutaneously injected into the right flank area of mice (female, four-week-old, six mice in each group). One week after injection, the tumor size was monitored twice weekly and tumor volume was calculated by following formula: V = π/6×L×W^2^ (where W was the widest diameter, and L was the perpendicular width) [26]. After 25 days, all mice were euthanized by intraperitoneal injection of 0.3% sodium pentobarbital (200 mg/kg; Merck, USA), and euthanasia was confirmed by cervical dislocation. Subsequently, tumor tissues were isolated from the mice and weighted. Additionally, the tumors were processed for Ki-67 staining and WB analysis following the relevant descriptions provided above. Animal experiments were approved by the Institutional Animal Care and Use Committee of Tongji Medical College, Huazhong University of Science and Technology (2022 IACUC Number 3159). The humane endpoint criteria were developed in consultation with veterinarians and animal welfare experts to ensure that any discomfort or distress experienced by the animals is minimized and mitigated. We have adhered to the highest standards of animal welfare and followed the ethical guidelines established by our institution in conducting these experiments.

### CCK-8 assay

The transfected breast cancer cells were plated into 96-well plates at a density of 3 × 10^3^ cells per well. 24 h after inoculation, cells were treated with C59 at 20 µmol/L for 24 h. Next, 10 µL of CCK-8 solution was added into each well, and incubated for 4 h. The absorbance at 450 nm was determined by microplate reader (Thermo, USA).

### Statistical analysis

SPSS 22.0 software was used to perform statistical analysis. Data were expressed as the mean ± standard deviations (SD). Graphpad Prism 8.04 software was applied to graph. For cell lines, statistical differences in two groups were determined by an unpaired Student’s t-test (two-tailed). The differences in CDCA5 expression between breast cancer tissues and normal tissues were analyzed using a paired Student’s t-test (two-tailed). Statistical analysis of more than two groups was evaluated by one‐way analysis of variance (ANOVA) followed by a Tukey post-hoc test. The paired sign test was used for statistical analysis of Table 1. For Table 2, the expression levels of CDCA5 and the clinicopathologic characteristics in the breast cancer samples were compared using non‑parametric tests (Mann-Whitney U test for comparison between two groups, and the Kruskall‑Wallis test for comparison between three or more groups). Correlation analysis in Figure S3B was conducted by Spearman correlation coefficient (r) test. Correlation analysis between CDCA5/E2F1/FOXM1 expression and overall survival (OS) presented in Figure S2 was completed by survMisc package, which provides the Renyi test inside. The Kaplan–Meier method was utilized to draw OS curve. The *p* value of < 0.05 was considered significantly difference.

**Table 1:** Expression patterns in breast cancer tissues and normal tissues were revealed by immunohistochemistry analysis

CDCA5 expression	Tumor tissue		Para-carcinoma tissue		*p* value
	Cases	Percentage	Cases	Percentage	
Low	48	50.0%	15	68.2%	< 0.001
High	48	50.0%	7	31.8%	

**Table 2:** Relationship between CDCA5 expression and clinicopathologic characteristics in patients with breast cancer

Features	No. of patients	CDCA5 expression		*p* value
		low	high	
All patients	96	48	48	
Age (years, 37 ~ 88)				0.542
<58	47	25	22	
≥ 58	49	23	26	
Grade				0.910
II	46	23	23	
III	41	20	21	
AJCC stage				0.063
1	1	1	0	
2	13	11	2	
3	50	23	27	
4	28	13	15	
T Infiltrate				< 0.05
0	1	1	0	
1	21	16	5	
2	59	26	33	
3	12	5	7	
lymphatic metastasis(N)				0.633
0	47	25	22	
1	24	11	13	
2	14	8	6	
3	10	4	6	
Tumor size				< 0.05
≤ 3 cm	53	33	20	
> 3 cm	40	15	25	
Lymph node positive				0.678
< 1	44	23	21	
≥ 1	48	23	25	
ER nuclear test results				0.463
< 6	45	20	25	
≥ 6	46	24	22	
PR nuclear test results				0.651
=0	53	27	26	
> 0	39	18	21	
HER2 testing results of membrane				0.688
≤ 6	49	23	26	
> 6	43	22	21	
Ki-67 nuclear test results				0.144
≤ 2	48	27	21	
> 2	44	18	26	
P53 nuclear test results				0.756
≤ 3	47	24	23	
> 3	46	22	24	
FISH data				0.773
negative	62	30	32	
positive	33	17	16	

**Table 3:** Spearman correlation analysis between CDCA5 expression and clinicopathologic characteristics in patients with breast cancer

		POLE2
Tumor size	Spearman correlation	0.245
	Significance (two-tailed)	< 0.05
	N	93
T Infiltrate	Spearman correlation	0.256
	Significance (two-tailed)	< 0.05
	N	93

## Results

### CDCA5 was upregulated in breast cancer and correlated with poor prognosis

**Fig. 1:**
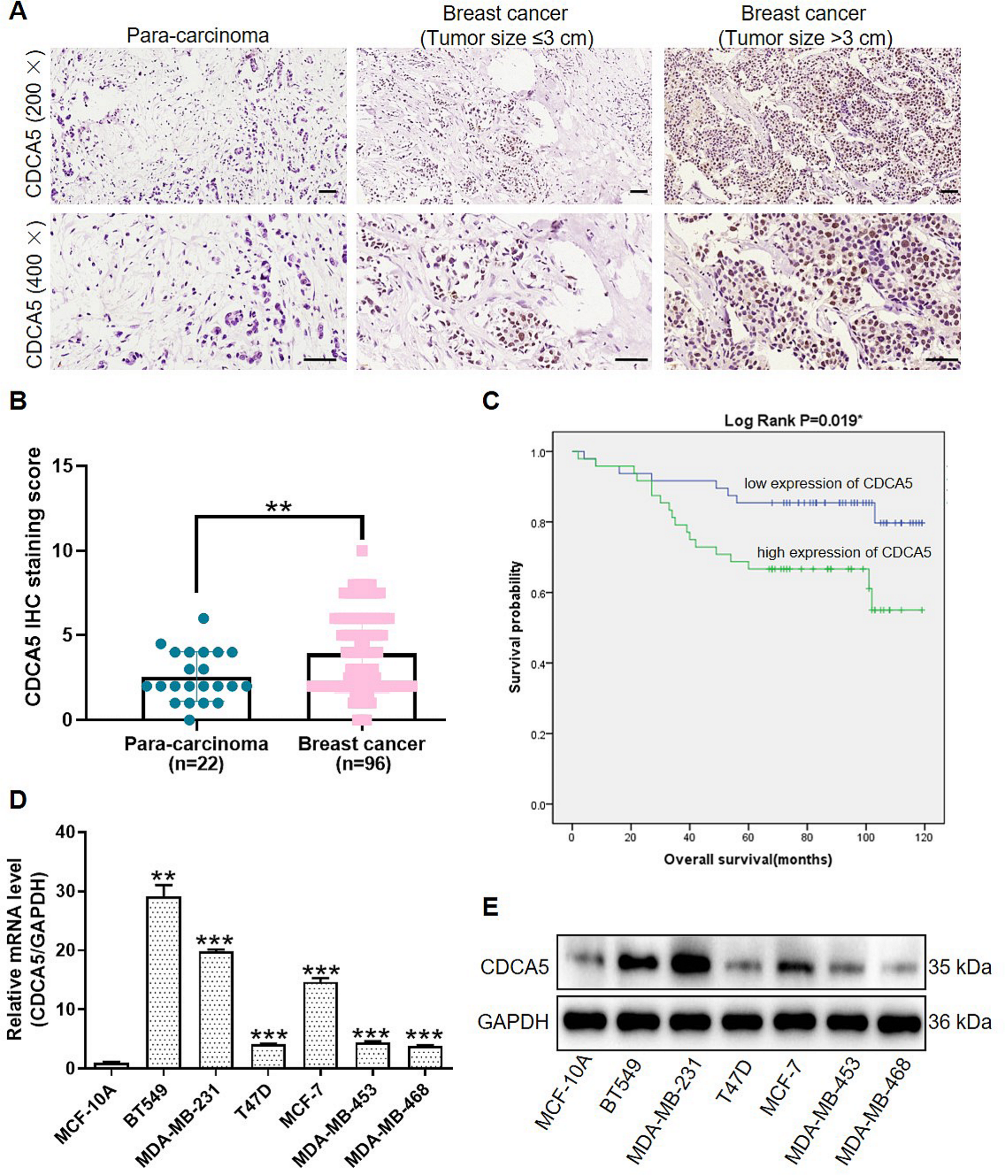
CDCA5 was upregulated in breast cancer and correlated with poor prognosis. **(A)** Representative IHC images of CDCA5 staining in human breast cancer tissues and normal para-carcinoma tissues. Scale bar is 50 μm. **(B)** The IHC staining score of CDCA5 in Fig. 1A. **(C)** Overall survival curves of breast cancer patients with high/low expression of CDCA5. High expression of CDCA5: 48 samples; Low expression of CDCA5: 48 samples. **(D)** CDCA5 mRNA and **(E)** protein expression levels in breast cancer cell lines (BT-549, MDA-MB-231, T47D, MCF-7, MDA-MB-453, MDA-MB-468) and the normal MCF-10 A cell line was detected by qPCR analysis and WB analysis. GAPDH was used as an internal reference. Results were presented as mean ± SD. ^*^p < 0.05

Firstly, we identified the expression of CDCA5 in human breast cancer tissues by IHC analysis of clinical breast cancer (*n* = 96) and para-carcinoma tissues (*n* = 22). The results of IHC staining indicated that expression of CDCA5 in breast cancer was higher than that in para-carcinoma tissues (Fig. 1A-B). Statistics verified the significant difference of CDCA5 overexpression in tumor tissues (50.0%) and normal tissues (31.8%) (*p* < 0.001) (Table 1). According to the Mann-Whitney U and Spearman correlation analysis, expression of CDCA5 was significantly associated with tumor size and T infiltrate (*p* < 0.05). However, no statistically significant correlation was observed between CDCAC5 expression and other clinicopathologic features, such as age, grade, stage, lymphatic metastasis, ER/PR/Ki-67/p53 nuclear test and HER2 membrane test (Table 2, and 3). In addition, we revealed that high expression of CDCA5 was correlated with poor overall survival (Fig. 1C). Consistently, CDCA5 expression was upregulated in various breast cancer cell lines, especially in BT-549, MDA-MB-231 and MCF-7 cells, compared to the normal MCF-10 A cells (Fig. 1D-E). These results indicated that the expression of CDCA5 was upregulated in breast cancer, and high CDCA5 expression was associated with poor prognosis of breast cancer.

### CDCA5 knockdown inhibited proliferation and migration of breast cancer cell in vitro

**Fig. 2:**
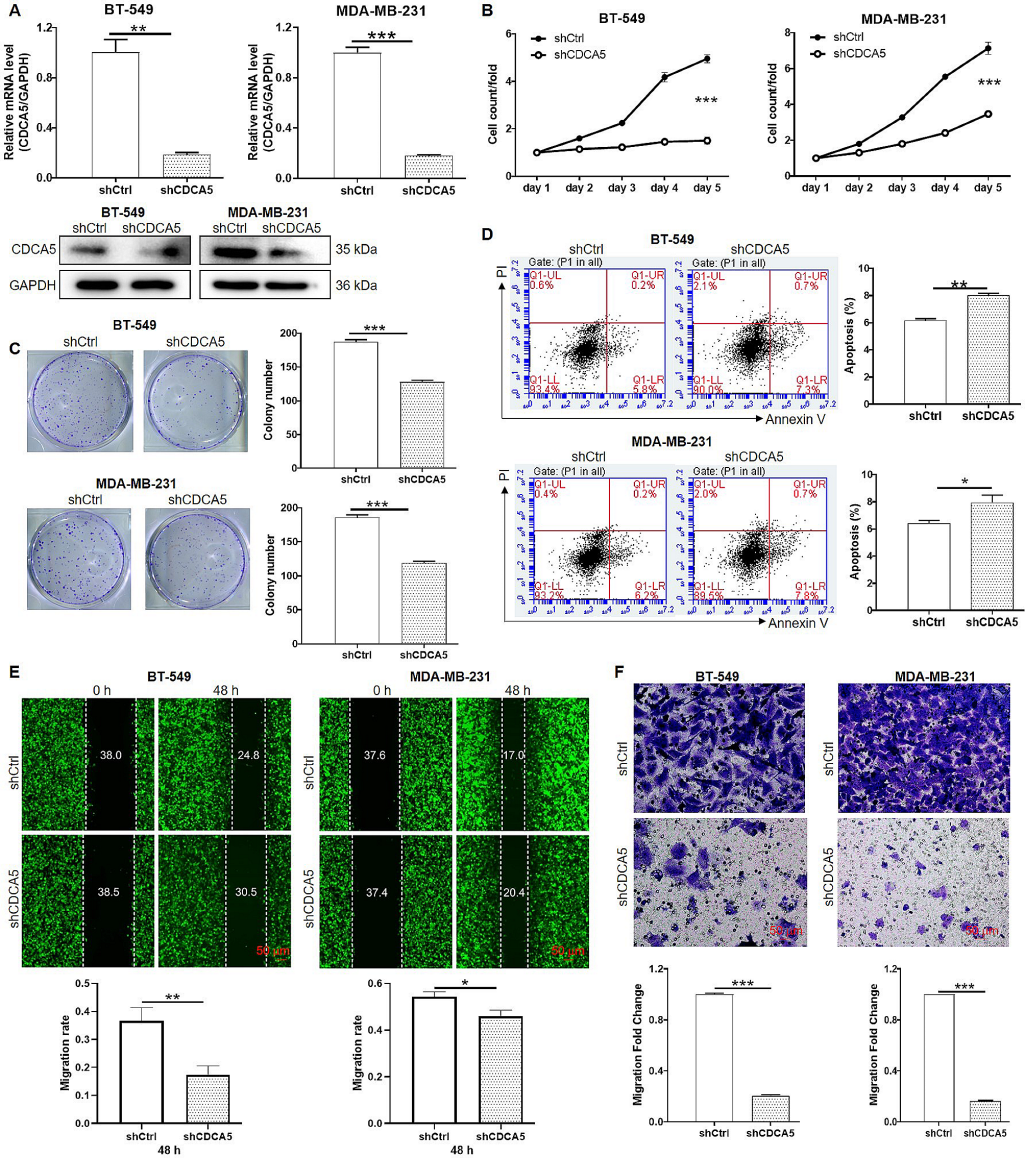
CDCA5 knockdown inhibited proliferation and migration of breast cancer cell in vitro. The BT-549 and MDA-MB-231 cell lines were transfected with shCDCA5 or shCtrl lentivirus. 48 h after lentiviral transfection, (**A**) the mRNA and protein levels of CDCA5 expression were evaluated by qPCR and WB assays, respectively. GAPDH was used as inner control. (**B**) Cell viability of BT-549 and MDA-MB-231 cells was determined by Celigo cell counting assay. (**C**) Capacity of colony formation in BT-549 and MDA-MB-231 cells was assessed by colony formation assays. (**D**) The apoptotic ratio of BT-549 and MDA-MB-231 cells were determined by flow cytometry. (**E**) Capacity of cell migration in BT-549 and MDA-MB-231 cells was detected by wound healing and (**F**) transwell assays. Results were presented as mean ± SD. ^*^p < 0.05, ^**^p < 0.01, ^***^p < 0.001

To evaluate the biological functions of CDCA5 in breast cancer cell proliferation and migration, the expression of CDCA5 was knocked down in BT-549 and MDA-MB-231 cell lines by according lentivirus. The shCDCA5-2 showed the most obvious knockdown efficiency (85.3%, *p* < 0.05) compared to shCDCA5-1 (84.0%, *p* < 0.05) and shCDCA5-3 (83.6%, *p* < 0.05) (Figure. S1A). Thus, shCDCA5-2 lentivirus was selected to knock down CDCA5 expression in two breast cancer cell lines. The qPCR and WB assays suggested a significant downregulation of CDCA5 at both mRNA and protein levels, indicating that CDCA5-knockdown cell models were successfully constructed (*p* < 0.01) (Fig. 2A). Proliferation of BT-549 and MDA-MB-231 transfected with shCtrl or shCDCA5 lentivirus was measured using Celigo cell count assay. As shown in Fig. 2B, cell growth in two cell lines with CDCA5 depletion were all inhibited relative to that in shCtrl group (*p* < 0.001). Consistently, the colony formation assay suggested that CDCA5 knockdown significantly decreased the capacity of colony formation of BT-549 and MDA-MB-231 cells (*p* < 0.001) (Fig. 2C). Furthermore, the apoptotic cell percentages in CDCA5-depleted breast cancer cells were largely increased versus that in shCtrl-transfected breast cancer cells (*p* < 0.001) (Fig. 2D). In wound healing assays, the migration distance of the cells was observed in microscope fluorescence imaging mode. The results suggested that BT-549 and MDA-MB-231 cells transfected with shCDCA5 lentivirus displayed impairment of migration ability when compared with cells transfected with shCtrl (*p* < 0.05) (Fig. 2E). Moreover, transwell assays were performed using Corning 3422 with 8 μm membrane pore size. By counting the migratory cells number under a microscope bright field, we found that CDCA5 knockdown indeed attenuated the migration ability of BT-549 and MDA-MB-231 cells (*p* < 0.001) (Fig. 2F). Collectively, these results suggested that CDCA5 knockdown dramatically inhibited the malignant proliferation, colony formation and migration capacities of breast cancer cells in vitro, while promoted cell apoptosis.

### CDCA5 promoted the binding of E2F1 to FOXM1 promoter

**Fig. 3:**
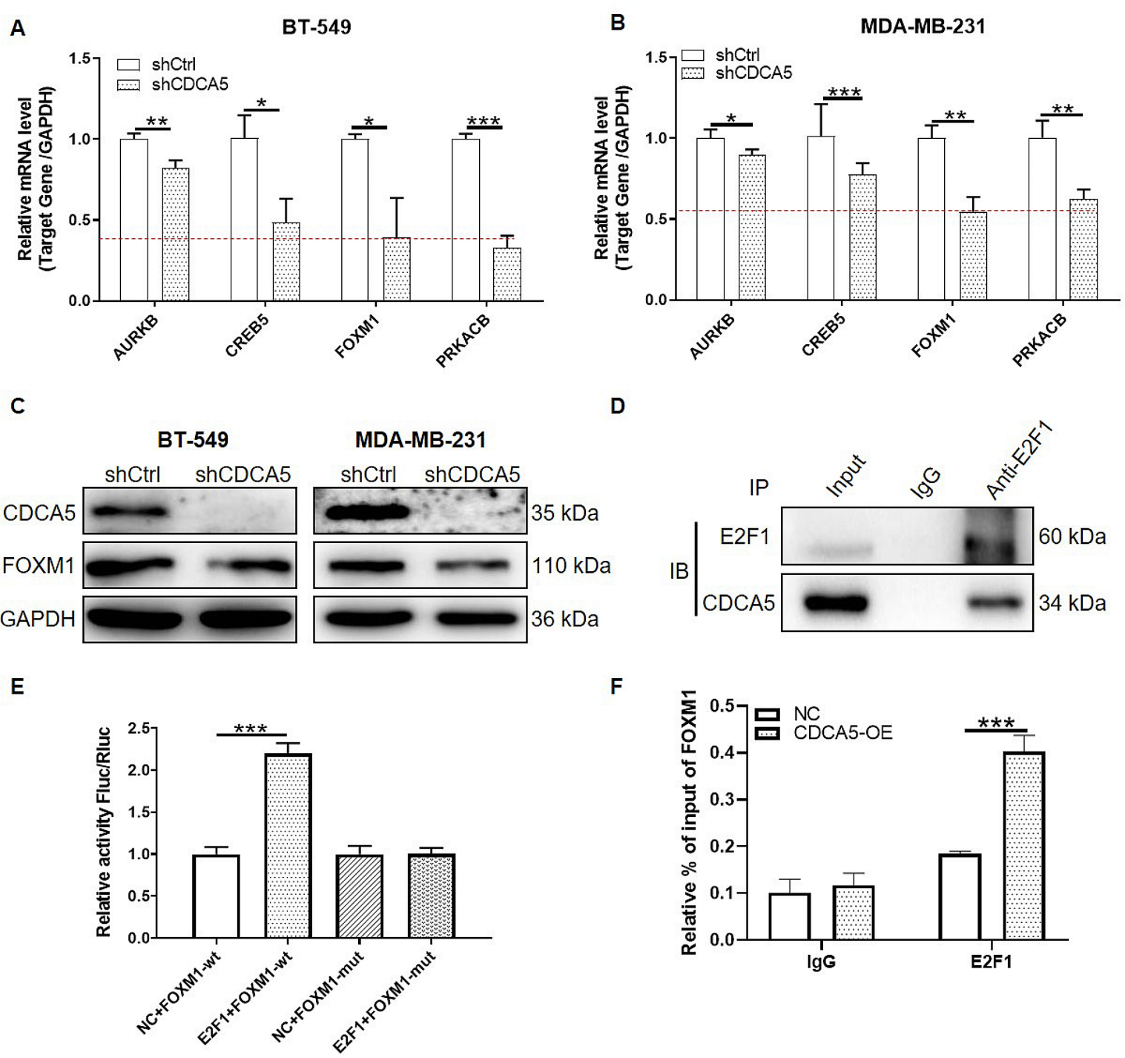
CDCA5 promoted the binding of E2F1 to FOXM1 promoter. **(A-B)** The mRNA levels of AURKB, CREB5, FOXM1, and PRKACB in BT-549 andMDA-MB-231 cells transfected with shCDCA5 or shCtrl lentivirus were determined by qPCR. The dotted line showed the expression of FOXM1 relative to other genes. **(C)** The protein levels of CDCA5 and FOXM1 in BT-549 and MDA-MB-231 cells transfected with shCDCA5 or shCtrl lentivirus were detected by WB assays. GAPDH was used as an internal reference. **(D)** The interaction between CDCA5 and E2F1 in MDA-MB-231 cells was validated by Co-IP assays. **(E)** The combination of E2F1 and FOXM1 promoter was determined by dual-luciferase reporter assays in 293T cells transfected with E2F1 or empty vector and WT or Mut FOXM1 promoter. **(F)** Enrichment of E2F1 at FOXM1 promoter region was observed by ChIP analysis in MDA-MB-231 cells with or without CDCA5 overexpression. Results were presented as mean ± SD. ^*^p < 0.05, ^**^p < 0.01, ^***^p < 0.001

To identify the potential CDCA5-targeted genes that were involved in the malignant behaviors of breast cancer cells, we observed the DEGs between shCDCA5-transfected and shCtrl-transfected MDA-MB-231 cells by human Gene Chip Prime View analysis. Compared to the cells transfected with shCtrl, 300 of genes were upregulated and 212 of genes were downregulated in cells with CDCA5 knockdown (Figure. S1B). By delineating the interaction network between CDCA5 and canonical AMPK signaling, ATM signaling, Breast cancer regulation by stathmin1 and senescence pathway, we identified several DEGs downstream of CDCA5 regulation. Notably, among these DEGs, such as AURKB, CREB5, FOXM1 and PRKACB emerged as a significant downstream gene influenced by CDCA5 (Figure. S1C). The qPCR analysis validated the significant downregulation of AURKB, CREB5, FOXM1 and PRKACB in BT-549 and MDA-MB-231 cells with CDCA5 knockdown (*p* < 0.05). Importantly, it’s worth noting that FOXM1 expression was relatively low in both cell types examined (Fig. 3A-B). Moreover, Accumulating evidence has revealed a significant overexpression of FOXM1 in various human cancers, including breast cancer [27, 28]. Our findings further supported this trend, demonstrating elevated levels of FOXM1 in breast cancer cells (MDA-MB-231, BT-549 and T47D) in comparison with normal MCF-10 A cells (*p* < 0.001) (Figure. S1D). Additionally, dysregulation of p53 and Rb-E2F has been identified as essential for the overexpression of FOXM1 across multiple cancer types, and knockdown or overexpression of E2F1 leads to a corresponding reduction or elevation in FOXM1 expression [29, 30]. Therefore, we focused on FOXM1, and speculated that CDCA5 regulated FOXM1 expression through E2F1. Our WB analysis suggested that the protein levels of FOXM1 was decreased apparently with CDCA5 depletion (Fig. 3C). Depending on the STRING database (http://string-db.org), we predicted that E2F1 was co-expressed with CDCA5 (Figure. S1E). Additionally, we obtained the RNAseq counts of breast cancer from TCGA database, analyzed the correlation between expression of CDCA5, E2F1, FOXM1 and breast cancer survival. The data revealed significant overexpression of CDCA5, E2F1, and FOXM1 in breast cancer (Figure. S2A), and the elevated expression of these genes individually, as well as their combined overexpression, was correlated with poor overall survival (Figure. S2B). Moreover, advanced-stage breast cancer exhibiting higher expression levels of CDCA5, E2F1, and FOXM1 compared to early-stage tumors (Figure. S3A). Correlation analysis demonstrated a positive correlation between CDCA5 and FOXM1 (Pearson *r* = 0.6577, *p* < 0.001), CDCA5 and E2F1 (Pearson *r* = 0.6343, *p* < 0.001), as well as FOXM1 and E2F1 (Pearson *r* = 0.6262, *p* < 0.001) (Figure. S3B). Therefore, we speculated that CDCA5, E2F1, FOXM1 play crucial roles in breast cancer progression, supported by the observed positive correlation among their expression levels in breast cancer. Subsequently, the Co-IP experiments in MDA-MB-231 cells revealed that CDCA5 was co-precipitated when endogenous E2F1 was pulled down by its antibody (Fig. 3D), suggesting that CDCA5 could interact with E2F1. To investigate whether CDCA5 regulated FOXM1 expression through binding to E2F1, we mutated the promoter region of FOXM1 and performed the dual-luciferase reporter assay to observe the effect of E2F1 on FOXM1 promoter activity. The results of dual-luciferase assay showed that luciferase activity in E2F1 and FOXM1-WT plasmids co-transfected cells was significantly increased compared to that in only FOXM1-WT plasmid transfected cells (*p* < 0.001). However, the expression of E2F1 was not able to increase luciferase activity of FOXM1-Mut, suggesting the presence of combination between E2F1 and FOXM1 promoter (Fig. 3E). In addition, by the ChIP assays, we verified that the E2F1 was recruited to the FOXM1 promoter region, and which was promoted by CDCA5 overexpression (Fig. 3F). Taken together, these data suggested that CDCA5 facilitated malignant behaviors of breast cancer cells via promoting the binding of E2F1 to FOXM1 promoter.

### FOXM1 depletion attenuated cell proliferation and migration promoted by CDCA5

**Fig. 4:**
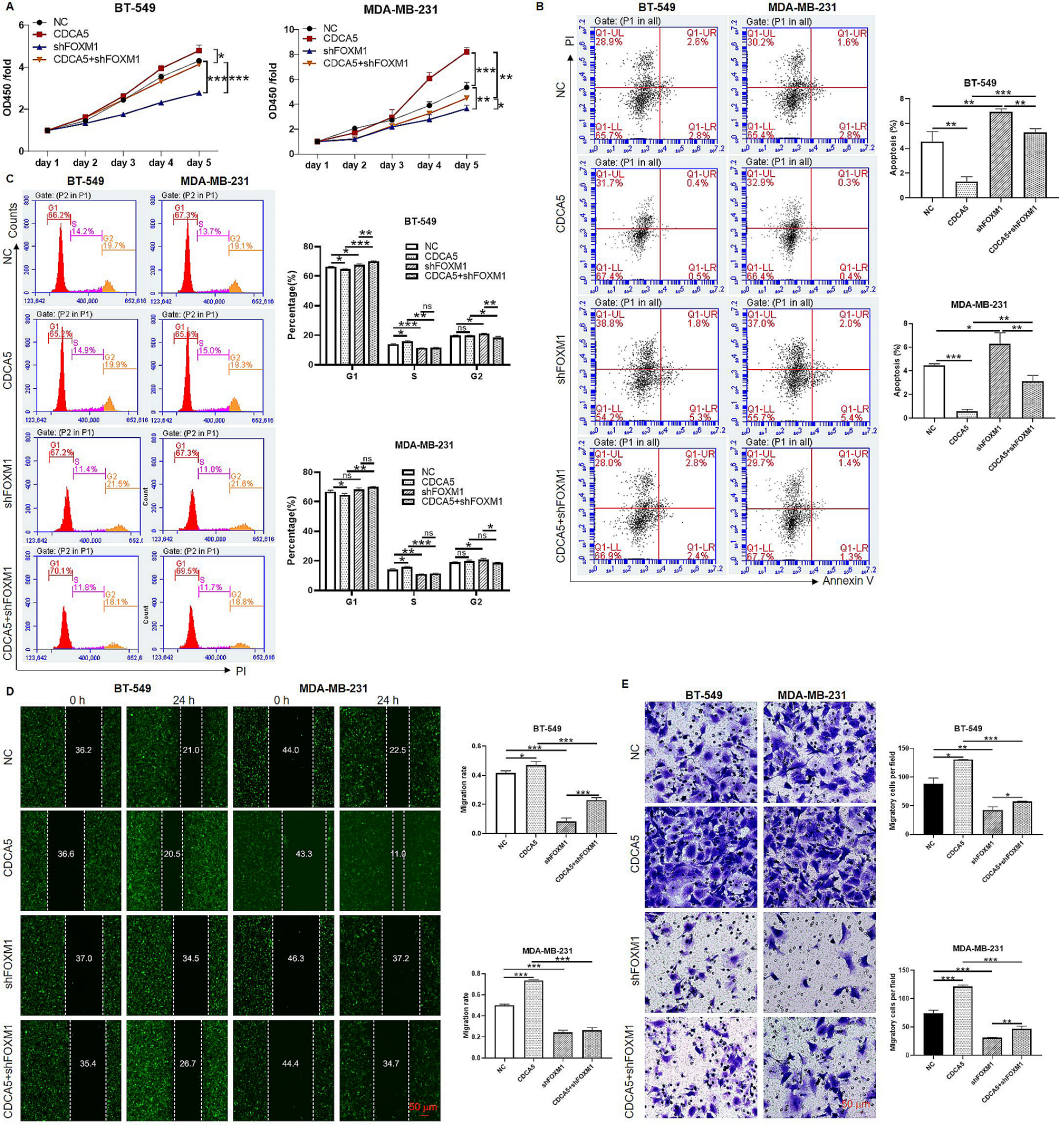
FOXM1 depletion attenuated cell proliferation and migration promoted by CDCA5. The BT-549 and MDA-MB-231 cell lines were transfected with CDCA5-overexpressed and FOXM1-depleted scramble vectors (NC group), CDCA5-overexpressed lentivirus and FOXM1-depleted scramble vector (CDCA5 group), CDCA5-overexpressed scramble vector and FOXM1-depleted lentivirus (shFOXM1 group), and CDCA5-overexpressed lentivirus and FOXM1-depleted lentivirus (CDCA5 + shFOXM1 group), respectively. 48 h after lentiviral transfection, **(A)** Cell viability in four groups of BT-549 and MDA-MB-231 cells was determined by Celigo cell counting assay. **(B)** The apoptotic ratio in four groups of BT-549 and MDA-MB-231 cells were evaluated by flow cytometry. **(C)** Cell cycle in four groups of BT-549 and MDA-MB-231 cells was analyzed by flow cytometry. **(D-E)** Capacity of cell migration in four groups of BT-549 and MDA-MB-231 cells was detected by wound healing and transwell assays. Results were presented as mean ± SD. ^*^p < 0.05, ^**^p < 0.01, ^***^p < 0.001

To validate the essential roles of FOXM1 in CDCA5 induced proliferation and migration of breast cancer cells, functional rescue experiments in BT-549 and MDA-MB-231 cells were performed by transfection of CDCA5 lentivirus, shFOXM1 lentivirus and co-transfection of CDCA5 and shFOXM1 lentivirus. We found a significant increase in cell viability in the CDCA5 group of BT-549 and MDA-MB-231 cells (*p* < 0.05), while cell viability was attenuated in the shFOXM1 and CDCA5 + shFOXM1 group compared to their respective control groups (*p* < 0.05) (Fig. 4A). Moreover, FOXM1 knockdown reversed the inhibitory effects of CDCA5 on cell apoptosis in both breast cancer cell lines (*p* < 0.01) (Fig. 4B). Consistently, the cell cycle assays in BT-549 and MDA-MB-231 showed that CDCA5 overexpression significantly increased the percentage of S-phase cells (*p* < 0.05) and decreased the percentage of G1-phase cells (*p* < 0.05), while it had no significant effect on the percentage of G2-phase cells. Compared with the CDCA5 overexpression group alone, the CDCA5 overexpression combined with FOXM1 knockdown group significantly decreased the proportion of S-phase cells (*p* < 0.01) and upregulated the proportion of G1-phase cells (*p* < 0.01) (Fig. 4C). In addition, by conducting wound healing and transwell assays, we found that overexpression of CDCA5 obviously increased cell migration capacities, whereas it was completely inhibited by downregulating FOXM1 (*p* < 0.05) (Fig. 4D-E). All together, we demonstrated that CDCA5 facilitated cell proliferation and migration, inhibited cell apoptosis via targeting FOXM1 in breast cancer.

### FOXM1 is essential for CDCA5-induced tumor growth in vivo

**Fig. 5:**
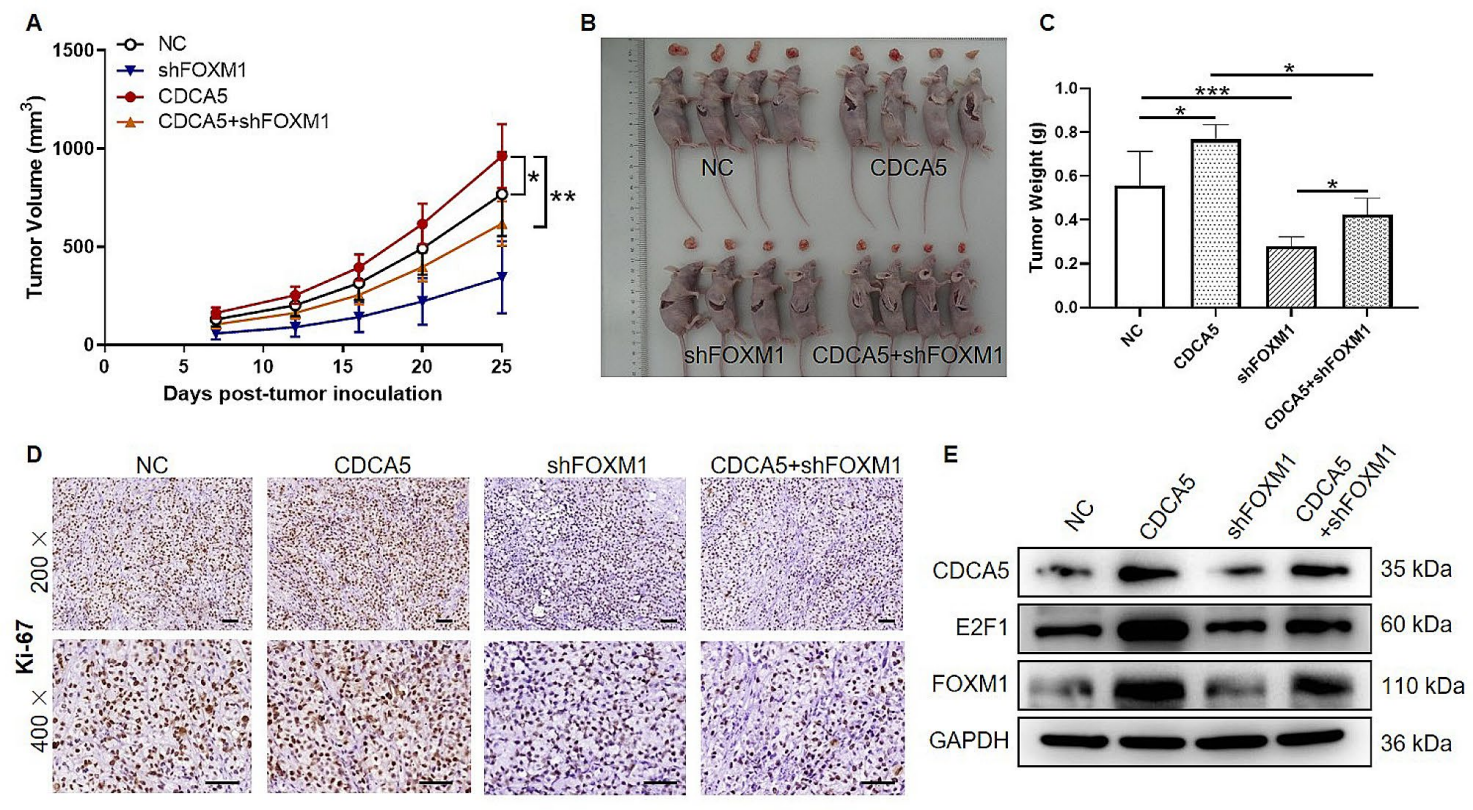
FOXM1 is essential for CDCA5-induced tumor growth in vivo. The xenograft model was constructed by subcutaneous injection of MDA-MB-231 cells from NC, shFOXM1, CDCA5 or CDCA5 + shFOXM1 groups. 7 days after inoculation, the tumor volume was calculated based on tumor sizes and **(A)** tumor growth curves were obtained by tumor volume. **(B)** Photographs of all mice and tumor tissues in four groups. **(C)** Tumor weight of mice in four groups. **(D)** Representative IHC images of Ki-67 staining in tumor tissues from mice in four groups. Scale bar is 50 μm. **(E)** The protein expression levels of CDCA5, E2F1 and FOXM1 in tumor tissues were determined by WB analysis. GAPDH served as an internal reference. Results were presented as mean ± SD. ^*^p < 0.05, ^**^p < 0.01, ^***^p < 0.001

We next evaluated the essential role of FOXM1 in CDCA5-induced tumor growth in vivo by subcutaneous injection of CDCA5-overexpressed, FOXM1-depleted, CDCA5-overexpressed + FOXM1-depleted or NC MDA-MB-231 cells. The tumor size was recorded continuously 7 days after inoculation, as shown in Fig. 5A, the tumor growth rate was significantly decreased in CDCA5 + shFOXM1 group as compared with that in CDCA5 (*p* < 0.01). After 25 days, the mice were sacrificed and tumor tissues were isolated. Representative pictures of both mice and tumor tissues indicated that FOXM1 knockdown impaired tumor growth of breast cancer cells induced by CDCA5 in vivo, which was validated by results of tumor weight (Fig. 5B-C). Additionally, we found that CDCA5 overexpression upregulated Ki-67 expression, while Ki-67 expression in shFOXM1 and CDCA5 + shFOXM1 group was all apparently reduced (Fig. 5D). In line with above results, the WB analysis in tumor tissues of mice suggested that the protein levels of FOXM1 and E2F1 were significantly upregulated in CDCA5-overexpressiong tumor tissues (Fig. 5E). These results demonstrated that the expression of FOXM1 was regulated by CDCA5 and CDCA5 triggered tumorigenicity of breast cancer cell in vivo through FOXM1.

### CDCA5 facilitated proliferation of breast cancer cells via Wnt/β-catenin signaling pathway

**Fig. 6:**
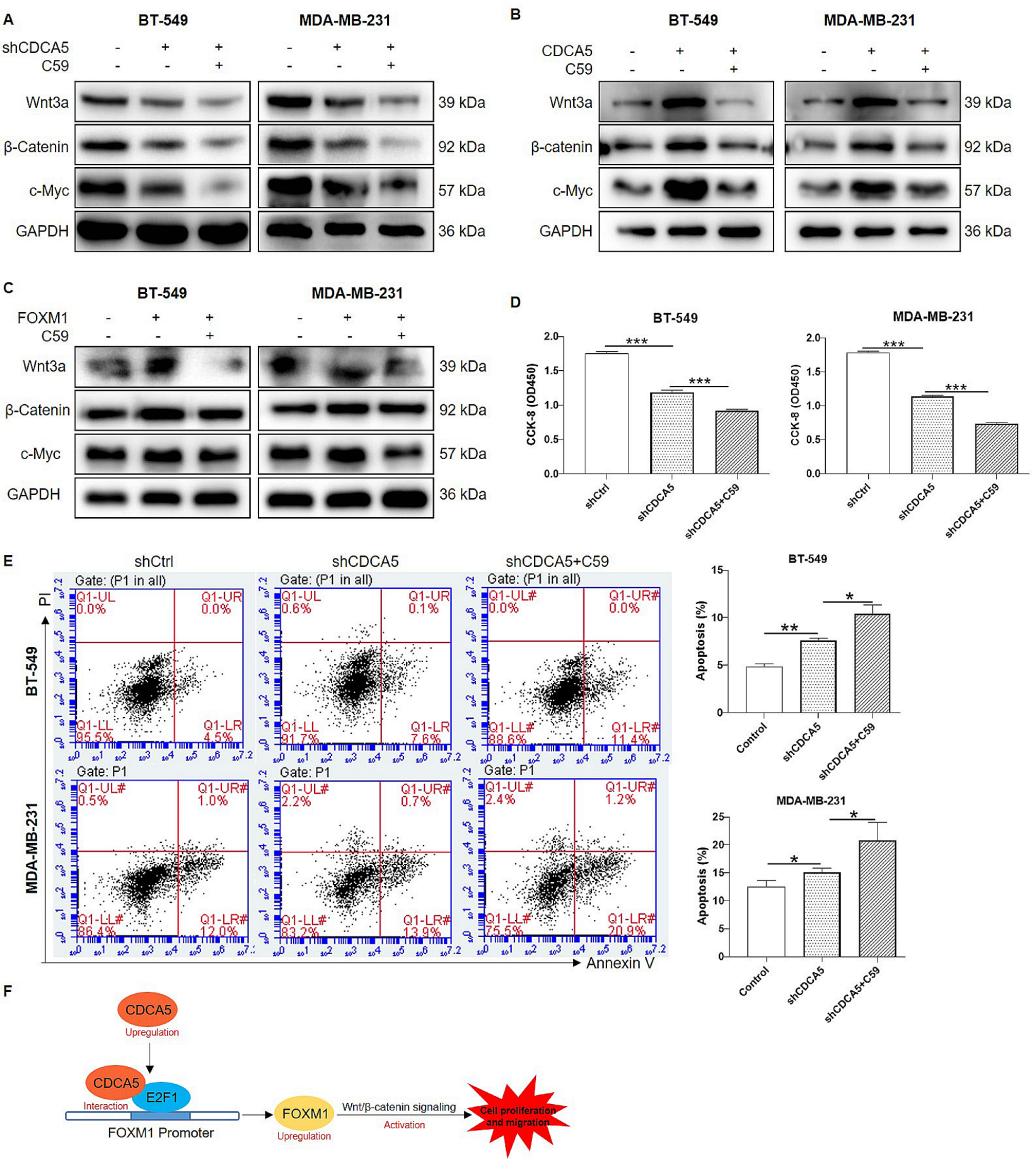
CDCA5 facilitated proliferation of breast cancer cells via Wnt/β-catenin signaling pathway. The BT-549 and MDA-MB-231 cell lines were transfected with indicated lentivirus followed by treatment of C59 (Wnt/β-catenin inhibitor, 20 µmol/L) for 24 h or not. **(A-C)** Wnt3a, β-catenin and c-Myc protein levels in three groups of BT-549 and MDA-MB-231 cells were detected by WB assays. GAPDH was used as an internal reference. **(D)** Cell viability in three groups of BT-549 and MDA-MB-231 cells were determined by CCK-8 assays. **(E)** The apoptotic ratio in three groups of BT-549 and MDA-MB-231 cells were analyzed by flow cytometry. **(F)** Schematic depiction illustrated that CDCA5 promoted Wnt/β-catenin signaling activation and breast cancer progression through upregulating FOXM1 transcription. Results were presented as mean ± SD. ^**^p < 0.01, ^***^p < 0.001

The Wnt/β-catenin is a canonical Wnt signaling pathway implicated in stem cell regeneration and cell survival [31]. Furthermore, Wnt activation has been observed in breast and associated with recurrence [32]. Notably, upregulation of FOXM1 has been reported to activate the Wnt/β-catenin signaling pathway, thus promoting the development of breast cancer [33]. Therefore, we assumed that Wnt/β-catenin signaling may be essential for CDCA5-mediated breast cancer progression. As shown in Fig. 6A, the results of WB assays in BT-549 and MDA-MB-231 cells suggested that C59, an inhibitor of Wnt/β-catenin, apparently enhanced the inhibitory function of shCDCA5 on protein expression of key proteins in the Wnt/β-catenin pathway, including Wnt3a, β-catenin, and the downstream target protein c-Myc. Moreover, compared to the control group, overexpression of CDCA5 or FOXM1 markedly upregulated the expression of Wnt3a, β-catenin and c-Myc, while treatment of C59 significantly attenuated the upregulation of Wnt3a, β-catenin, and c-Myc induced by CDCA5 or FOXM1 overexpression (Fig. 6B-C). These findings suggest that the CDCA5/FOXM1 axis may indeed activate the Wnt/β-catenin signaling pathway in breast cancer. In addition, cell viability in shCDCA5 + C59 group of BT-549 and MDA-MB-231 cells was significantly decreased as compared with that in shCDCA5 group (Fig. 6D). In comparison, inhibition of Wnt/β-catenin induced by C59 promoted the cell apoptosis obviously relative to shCDCA5 group (Fig. 6E). Altogether, these results indicated that Wnt/β-catenin signaling pathway served as the possible downstream of CDCA5 in breast cancer development.

## Discussion

Breast cancer is a leading cause of cancer related death in women worldwide [34]. In the past two decades, the mortality rate of breast cancer in numerous countries were keeping increasing [35]. However, traditional therapy regimens of surgery, radiotherapy and chemotherapy for breast cancer have limited effects on patients with advanced breast cancer [36, 37]. It is reported that the combination therapy, such as combination of immune checkpoint inhibitors and targeted agents showed great therapeutic effect that can effectively prolong the survival of breast cancer patients [38, 39]. However, most of the existing targeted agents for breast cancer have the limitations of high recurrence rates [38]. Therefore, we here aimed to unveil the pathogenesis of breast cancer and searched for a novel therapeutic target to improve patient outcomes.

CDCA5 was initially identified as a substrate of the anaphase-promoting complex, playing an essential role in regulating the cell cycle [10]. Additionally, CDCA5 has been implicated in several tumor progressions, including bladder cancer, hepatocellular carcinoma, gastric cancer and esophageal squamous cell carcinoma [12–16]. Importantly, CDCA5 expression was also found to be upregulated in patients with breast cancer and the breast cancer cell lines. In an agreement, we confirmed that CDCA5 was significantly overexpressed in human breast cancer tissues and cell lines as compared with corresponding controls. Moreover, recent studies suggested that overexpression of CDCA5 was an indicator of poor prognosis of patients with hepatocellular carcinoma [20, 40] and breast cancer [21]. Consistently, we revealed that upregulation of CDCA5 was associated with tumor size and tumor metastasis in breast cancer patients, as well as the poor survival. These findings indicated the possibility of CDCA5 as a promising target for breast cancer therapy.

On the other hand, it was demonstrated that CDCA5 knockdown could dramatically inhibit cell proliferation, migration and in vivo tumorigenesis in breast cancer [17, 18]. Additionally, CDCA5 was also involved in cell cycle control in breast cancer [21]. Similarly, our results indicated that CDCA5 knockdown significantly decreased cell viability, colony formation, cell migration and promoted cell apoptosis in breast cancer. CDCA5 overexpression led to an increase of S-phase cells proportion. Therefore, we showed that CDCA5 acted as a tumor promoter in breast cancer progression. However, the underlying mechanism by which how CDCA5 facilitates breast cancer remains unclear.

To further uncover the mechanism of CDCA5 contributing to breast cancer progression, the RNA-Seq analysis was performed using CDCA5-depleted and CDCA5-control cell specimens. The data suggested that FOXM1 was one of co-expressed with CDCA5 and was apparently downregulated at both mRNA and protein levels upon CDCA5 knockdown. Moreover, FOXM1 was identified as a potential downstream target of CDCA5 by analysis of IPA interaction network. Forkhead box M1 (FOXM1) is a member of the Forkhead box (Fox) protein family characterized by a master regulator of cell survival, self-renewal, and tumorigenesis in various cancer cells [41]. FOXM1 is well known to be a transcription factor that upregulated in a plethora of tumors and targeting FOXM1 is considered as a promising therapeutic strategy for human solid cancers [42]. In breast cancer, FOXM1 has been observed at elevated levels and is known to promote breast cancer cell stemness and migration in a YAP1-dependent manner [43]. Moreover, increased expression of FOXM1 has been associated with a poor prognosis for patients with breast cancer [44, 45], aligning with our results of bioinformatics. The transcription of FOXM1 was regulated by several transcription factors, such as cAMP responsive element-binding protein (CREB), signal transducer and activator of transcription 3 (STAT3), CCCTC-binding factor (CTCF), as well as E2F can interact directly with binding sites of FOXM1 to upregulate FOXM1 expression [46–49]. By STRING database, we found the potential protein interactions between CDCA5 and E2F1, and which was validated by further Co-IP experiments in MDA-MB-231 cells. In addition, we found that the E2F1 was recruited to the FOXM1 promoter region, indicating that CDCA5 was capable to affect FOXM1 expression via binding to E2F1, thereby promoting in the progression of breast cancer. It is worth noting that Chen et al. found that E2F1 can also induce the expression of CDCA5 [13]. Combined with our research results, CDCA5 may upregulate FOXM1 transcription by interacting with E2F1. Therefore, we also speculate that the interaction between CDCA5, E2F1, and FOXM1 may form a regulatory feedback loop. High expression of CDCA5 promotes FOXM1 expression through E2F1, and high expression of FOXM1 further induces upregulation of CDCA5 expression. This regulatory model warrants further investigation and validation.

Additionally, FOXM1 was reported to activate Wnt/β-catenin signaling pathway in breast cancer, thus promoted proliferation, invasion and epithelial-mesenchymal transition of breast cancer cells [33]. However, whether Wnt/β-catenin signaling was required for CDCA5 mediated breast cancer progression was still unclear. Herein, we found that the protein expression of Wnt3a, β-catenin and the targeted protein c-Myc was decreased along with CDCA5 depletion, and which was enhanced by C59, an inhibitor of Wnt/β-catenin signaling. Moreover, the inhibition of cell viability or promotion of apoptosis induced by CDCA5 knockdown was more obvious in the presence of C59. Therefore, we showed that Wnt/β-catenin signaling may serve as the possible downstream of FOXM1 in breast cancer progression.

In summary, we identified that CDCA5 was significantly upregulated in breast cancer and it predicted poor survival of breast cancer patients. Especially, CDCA5 promoted proliferation, migration and inhibited apoptosis of breast cancer cells, which was alleviated by silencing FOXM1. The promoting role of CDCA5 was achieved by E2F1 transcriptional regulation of FOXM1 and activation of Wnt/β-catenin signaling (Fig. 6F). Therefore, we suggested that CDCA5 promoted progression of breast cancer via CDCA5/FOXM1/Wnt axis for the first time, CDCA5 may serve as a novel therapeutic target for breast cancer treatment. However, the role of CDCA5 was not investigated in the context of interactions with immune cells. This limitation underscores the necessity for future studies to explore the role of CDCA5 independent of immune cell involvement.

### Electronic supplementary material

Below is the link to the electronic supplementary material.

Supplementary Fig. 1. Knockdown efficacy and screening of potential downstream of CDCA5. (A) Transfection efficacy of three shCDCA5 lentivirus in MDA-MB-231 cells was determined by qPCR analysis. (B) The Hierarchical Clustering analysis of DEGs between CDCA5-depleted MDA-MB-231 cells and control cells. Red represented genes up-regulated, green represented genes downregulated. (C) The interaction network of CDCA5 and molecules in AMPK signaling, ATM signaling, Breast cancer regulation by stathmin1, Senescence pathway. (D) FOXM1 mRNA expression in breast cancer cell lines (MDA-MB-231, BT-549 and T47D) and the normal MCF-10 A cell line was detected by qPCR analysis. (E) STRING analysis revealed protein interactions between CDCA5 and its potential transcription factors, including ATM, E2F1, FLT1, FOXO3, MYC, SP1 and TP53. The colored nodes represented query proteins and first shell of interactions. Line color indicated the type of interaction evidence. Results were presented as mean ± SD. *p < 0.05, ***p < 0.001

Supplementary Material 2

Supplementary Material 3

Supplementary Material 4

Supplementary Fig. 2. Clinical information analysis of TCGA-BRCA samples. (A) CDCA5, E2F1 and FOXM1 gene expression in human BRCA tissues (*n* = 1095) and normal solid tissues (*n* = 113) from TCGA. (B) Overall survival in TCGA-BRCA patients with high and low expression level of CDCA5, E2F1 and FOXM1 genes alone or in combination

Supplementary Fig. 3. Clinical information analysis of TCGA-BRCA samples and correlation analysis between CDCA5, E2F1, FOXM1. (A) CDCA5, E2F1 and FOXM1 gene expression in the TCGA-BRCA tissues with different tumor stage. (B) Pearson correlation analysis between CDCA5, E2F1 and FOXM1 expression

Supplementary Material 7

## Data Availability

All data generated or analysed during this study are included in this published article and these data are available from corresponding author on reasonable request.

## Abbreviations

CDCA5: Cell division cycle associated 5
E2F1: E2F transcription factor 1
FOXM1: Forkhead box M1
TNBC: Triple-negative breast cancer
TCGA: The Cancer Genome Atlas
IHC: Immunohistochemical
DAB: Diaminobenzene
ATCC: American Type Culture Collection
FBS: Fetal bovine serum
qPCR: Real-time quantitative PCR
cDNA: Complementary DNA
SDS-PAGE: Sodium dodecyl sulfate polyacrylamide gel electrophoresis
PVDF: Polyvinylidene difluoride
HRP: Horseradish peroxidase
IPA: Ingenuity Pathway Analysis
Co-IP: Co-immunoprecipitation
SD: Standard deviations
OS: Overall survival
Fox: Forkhead box
CREB: Element-binding protein
STAT3: Signal transducer and activator of transcription 3
CTCF: CCCTC-binding factor
