# Supplementary figures and images for: CDCA5 accelerates progression of breast cancer by promoting the binding of E2F1 and FOXM1
Source: J Transl Med. 2024 Jul 8;22:639. doi: 10.1186/s12967-024-05443-w (PMC11232132; doi:10.1186/s12967-024-05443-w)

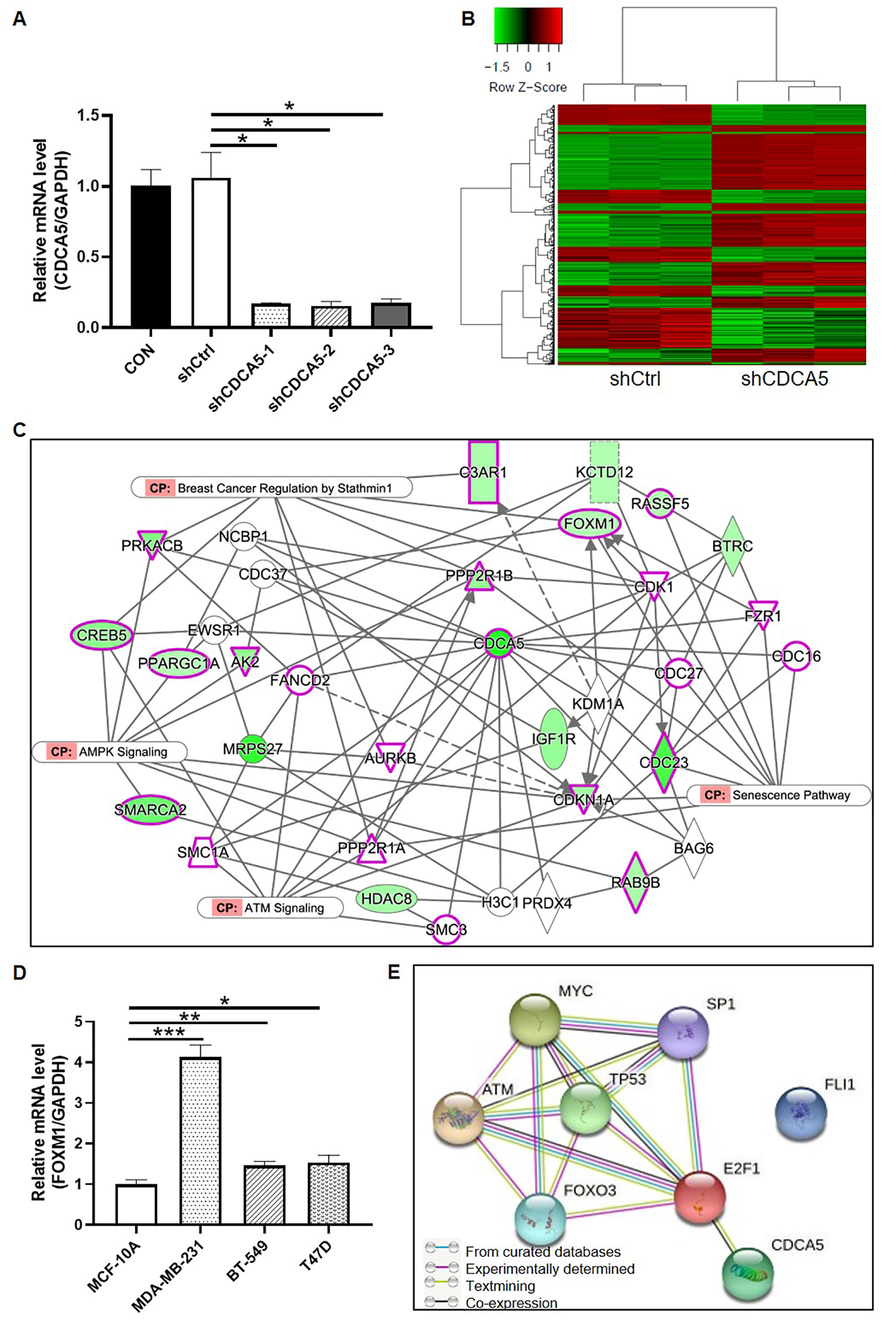

Supplement: Supplementary file 1 — Supplementary Fig. 1. Knockdown efficacy and screening of potential downstream of CDCA5. (A) Transfection efficacy of three shCDCA5 lentivirus in MDA-MB-231 cells was determined by qPCR analysis. (B) The Hierarchical Clustering analysis of DEGs between CDCA5-depleted MDA-MB-231 cells and control cells. Red represented genes up-regulated, green represented genes downregulated. (C) The interaction network of CDCA5 and molecules in AMPK signaling, ATM signaling, Breast cancer regulation by stathmin1, Senescence pathway. (D) FOXM1 mRNA expression in breast cancer cell lines (MDA-MB-231, BT-549 and T47D) and the normal MCF-10 A cell line was detected by qPCR analysis. (E) STRING analysis revealed protein interactions between CDCA5 and its potential transcription factors, including ATM, E2F1, FLT1, FOXO3, MYC, SP1 and TP53. The colored nodes represented query proteins and first shell of interactions. Line color indicated the type of interaction evidence. Results were presented as mean ± SD. *p < 0.05, ***p < 0.001 [file 12967_2024_5443_MOESM1_ESM.tif]

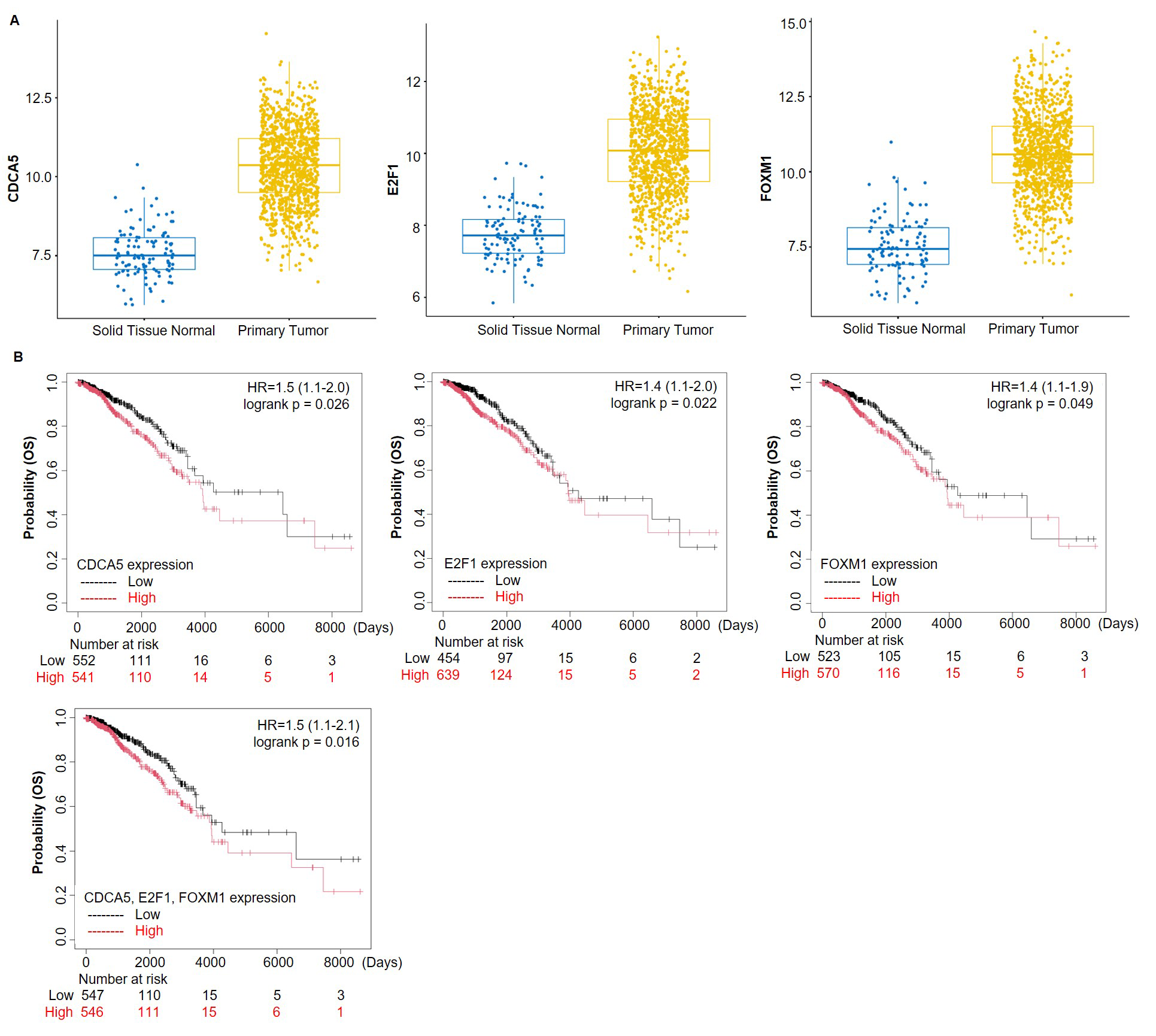

Supplement: Supplementary file 5 — Supplementary Fig. 2. Clinical information analysis of TCGA-BRCA samples. (A) CDCA5, E2F1 and FOXM1 gene expression in human BRCA tissues (n = 1095) and normal solid tissues (n = 113) from TCGA. (B) Overall survival in TCGA-BRCA patients with high and low expression level of CDCA5, E2F1 and FOXM1 genes alone or in combination [file 12967_2024_5443_MOESM5_ESM.tif]

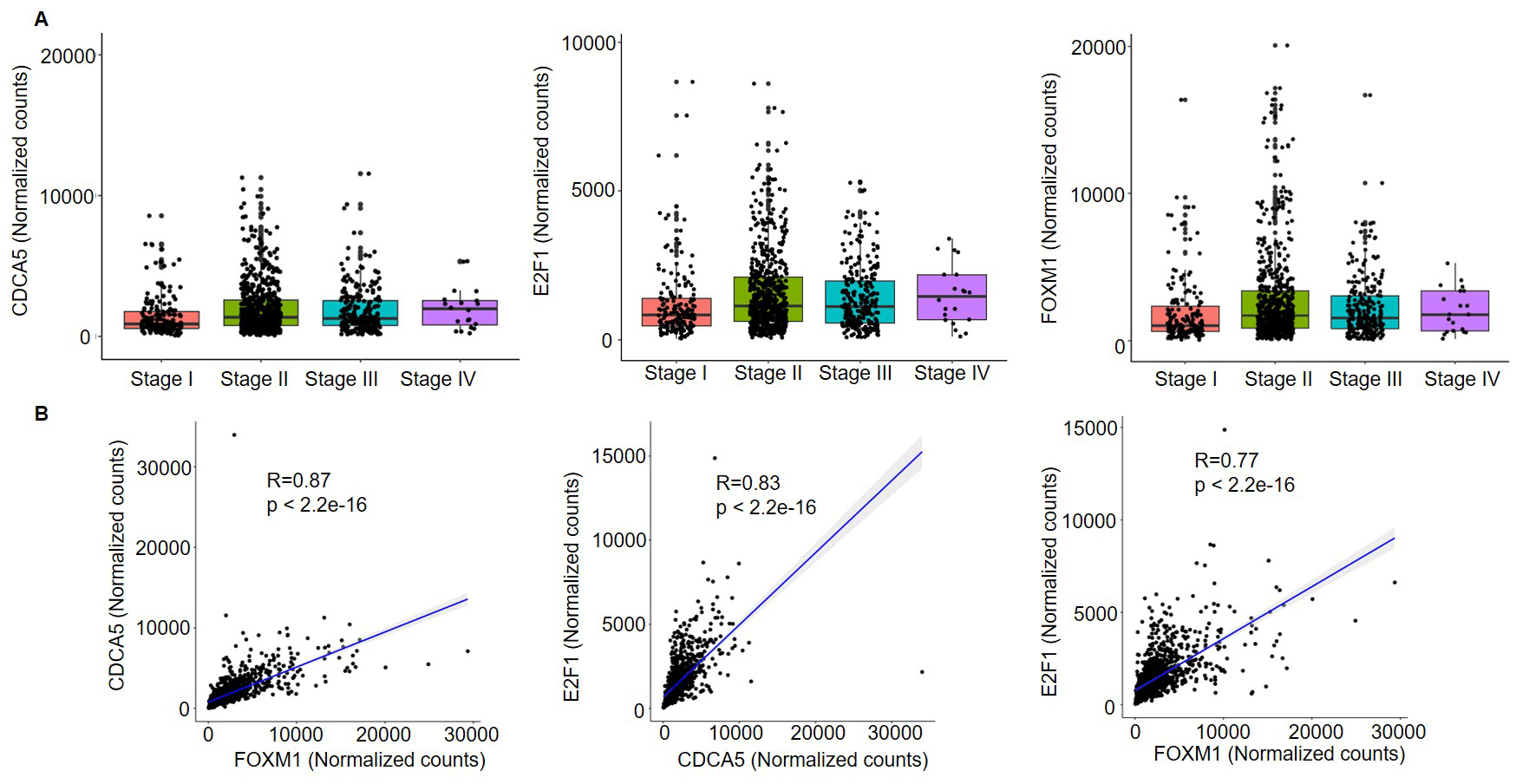

Supplement: Supplementary file 6 — Supplementary Fig. 3. Clinical information analysis of TCGA-BRCA samples and correlation analysis between CDCA5, E2F1, FOXM1. (A) CDCA5, E2F1 and FOXM1 gene expression in the TCGA-BRCA tissues with different tumor stage. (B) Pearson correlation analysis between CDCA5, E2F1 and FOXM1 expression [file 12967_2024_5443_MOESM6_ESM.tif]
