# Supplementary material for: CDCA5 accelerates progression of breast cancer by promoting the binding of E2F1 and FOXM1
Source: J Transl Med. 2024 Jul 8;22:639. doi: 10.1186/s12967-024-05443-w (PMC11232132; doi:10.1186/s12967-024-05443-w)
Supplement: Supplementary file 2 — Supplementary Material 2 [file 12967_2024_5443_MOESM2_ESM.docx]

**Supplementary Tables**

**Table S1** Target sequences of shRNAs

| Gene | No. | Target sequence (5'-3') |
| --- | --- | --- |
| CDCA5 | shCDCA5 -1 | TCCCTGAAATCTGGCCGAAGA |
| CDCA5 | shCDCA5 -2 | GTCAGAAAGCCCATCGTCTTA |
| CDCA5 | shCDCA5-3 | TGCGGAGGTCCCAGCGGAAAT |
| FOXM1 | shFOXM1-1 | AACATCAGAGGAGGAACCTAA |
| FOXM1 | shFOXM1-2 | ATGCTAATATTCACAGCATCA |
| FOXM1 | shFOXM1-3 | CAAGGAAGTGGCAGAGTCCAA |
