# Supplementary material for: CDCA5 accelerates progression of breast cancer by promoting the binding of E2F1 and FOXM1
Source: J Transl Med. 2024 Jul 8;22:639. doi: 10.1186/s12967-024-05443-w (PMC11232132; doi:10.1186/s12967-024-05443-w)
Supplement: Supplementary file 3 — Supplementary Material 3 [file 12967_2024_5443_MOESM3_ESM.docx]

**Table S2** Primers used in qPCR and ChIP assays.

| Primer name | Forward primer sequence (5’-3’) | Reverse primer sequence (5’-3’) |
| --- | --- | --- |
| CDCA5 | CCGAGCATCCTCCCTGAAAT | CATGGGCCACGATCCTCTTTA |
| AURKA | GGCACCTGAAAATAATCCTGAG | CAAAGTCTTCCAAAGCCCACT |
| CREB5 | GCCATGCAGAAAGAATCACAAG | TCGCTGACCGATGAGGAAGT |
| FOXM1 | AGTTCCCGGTGAACCAGTCA | ACACCACCTGTTCCCCAAA |
| PRKACB | TGGATTGGTGGGCATTAGG | GAACTGAAGTGGGATGGGAAT |
| FOXM1 promoter | GAAAGCTCCGGTGCCAGA | CTCCAACCTGGGGGCCGA |
| GAPDH | TGACTTCAACAGCGACACCCA | CACCCTGTTGCTGTAGCCAAA |
