# Supplementary material for: CDCA5 accelerates progression of breast cancer by promoting the binding of E2F1 and FOXM1
Source: J Transl Med. 2024 Jul 8;22:639. doi: 10.1186/s12967-024-05443-w (PMC11232132; doi:10.1186/s12967-024-05443-w)
Supplement: Supplementary file 4 — Supplementary Material 4 [file 12967_2024_5443_MOESM4_ESM.docx]

**Table S3** Antibodies used in IHC, WB, Co-IP and ChIP assays.

| Antibody | Diluted multiples/Dosage | Company | Catalog No. | Use |
| --- | --- | --- | --- | --- |
| CDCA5 | 1:100 | Abcam | Ab192237 | IHC |
| Ki-67 | 1:100 | Abcam | Ab16667 | IHC |
| CDCA5 | 1:1000 | CST | 58507S | WB, Co-IP |
| FOXM1 | 1:1000 | CST | 20459S | WB |
| Wnt3a | 1:500 | Abcam | Ab28472 | WB |
| c-Myc | 1:1000 | CST | 13987 | WB |
| β-Catenin | 1:5000 | Proteintech | 66379-1-lg | WB |
| GAPDH | 1:3000 | Proteintech | 60004-1-lg | WB |
| Goat anti-Rabbit | 1:3000 | Beyotime | A0208 | IHC, WB, Co-IP |
| Goat anti-Mouse | 1:3000 | Beyotime | A0216 | WB, Co-IP |
| Normal Rabbit IgG | 2 μg | CST | 13987S | ChIP |
| Histone H3 (D2B12) XP® Rabbit mAb | 2 μg | CST | 4620 | ChIP |
| E2F1 | 1:50 | SANTA CRUZ | Sc-251 | Co-IP, ChIP |
