# Supplementary material for: CDCA5 accelerates progression of breast cancer by promoting the binding of E2F1 and FOXM1
Source: J Transl Med. 2024 Jul 8;22:639. doi: 10.1186/s12967-024-05443-w (PMC11232132; doi:10.1186/s12967-024-05443-w)
Supplement: Supplementary file 7 — Supplementary Material 7 [file 12967_2024_5443_MOESM7_ESM.pptx]

## Slide 1
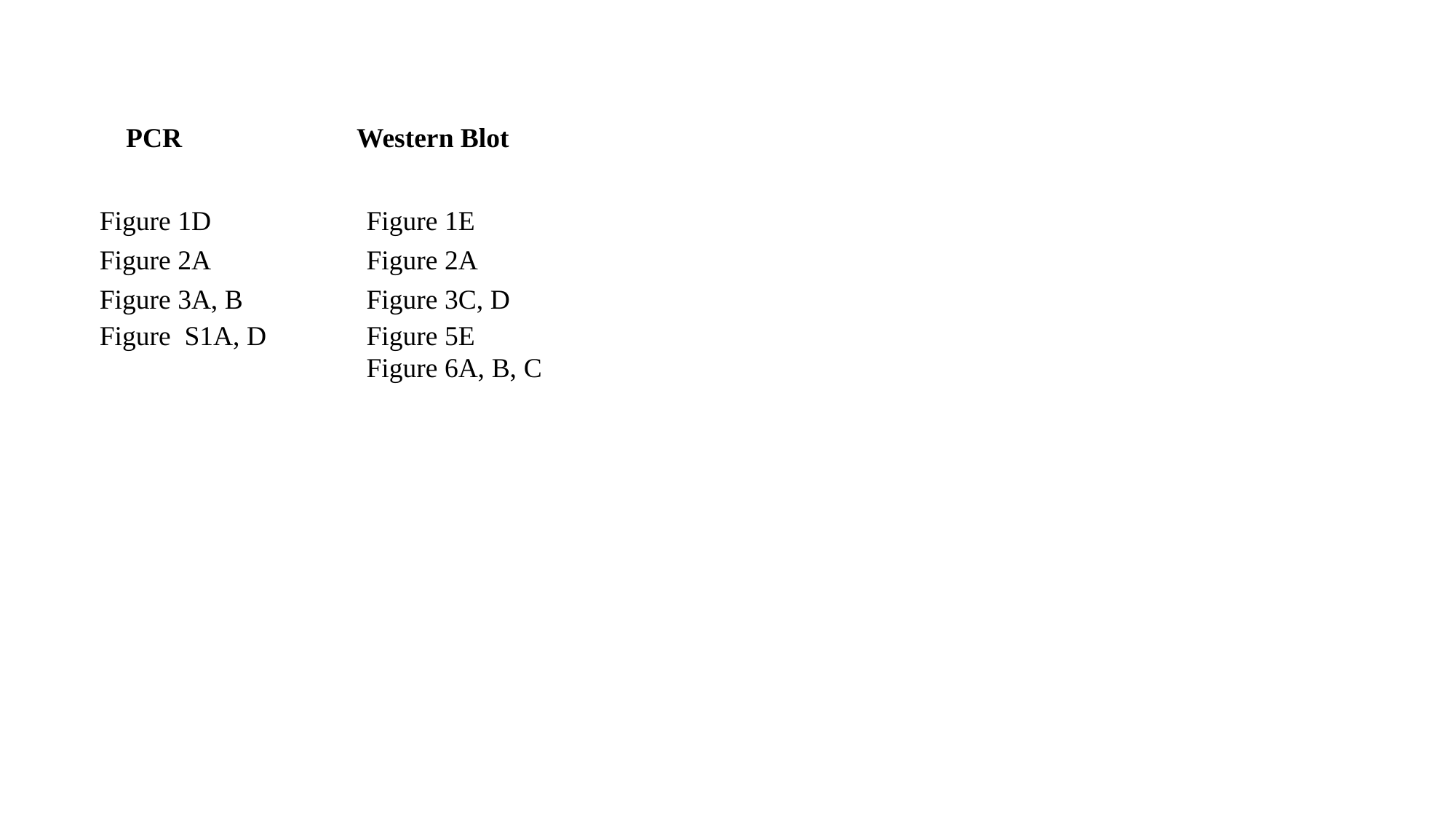

PCR
Western Blot
Figure 1D
Figure 1E
Figure 2A
Figure 2A
Figure 3A, B
Figure 3C, D
Figure S1A, D
Figure 5E
Figure 6A, B, C

## Slide 2
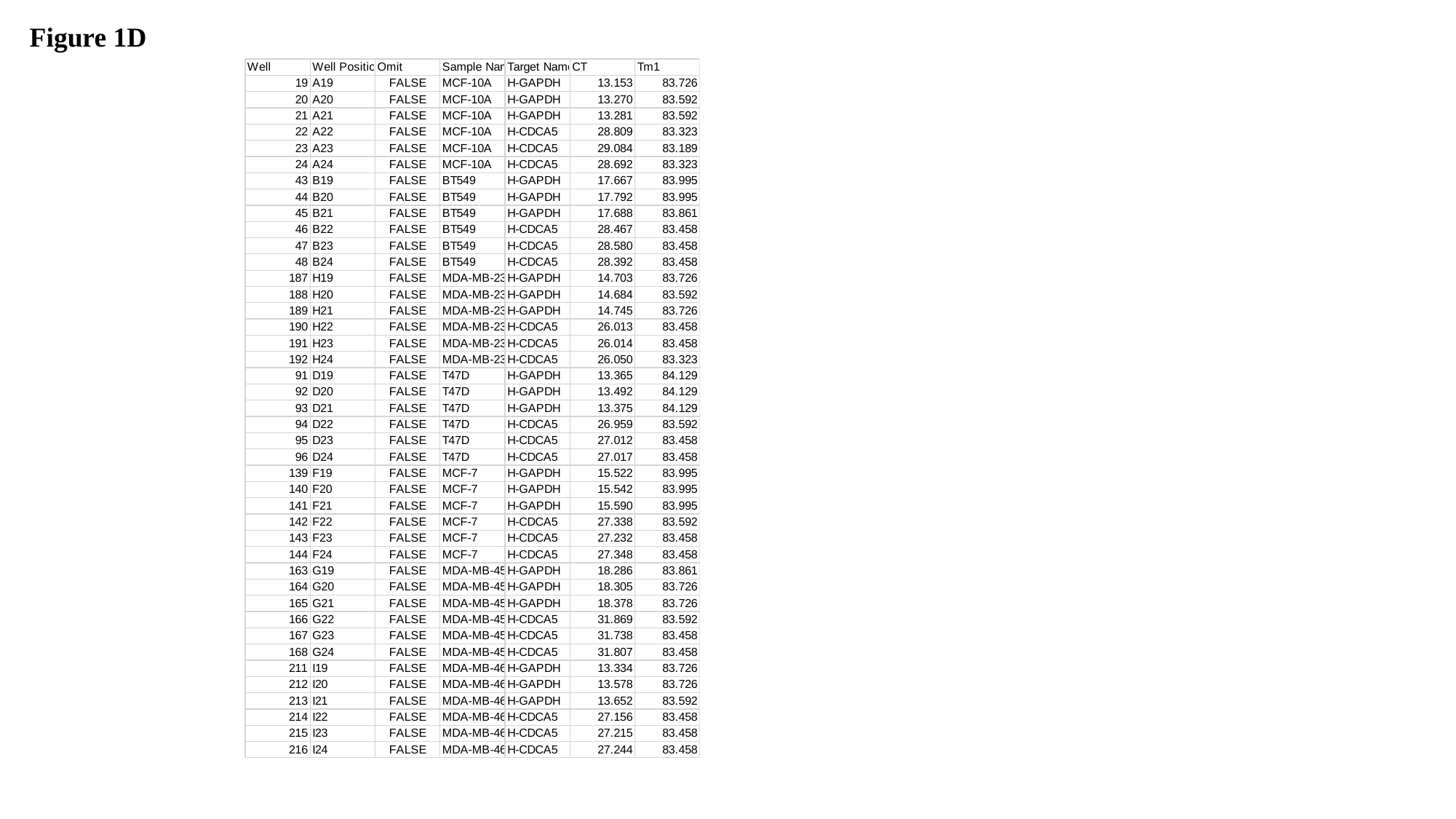

Figure 1D

## Slide 3
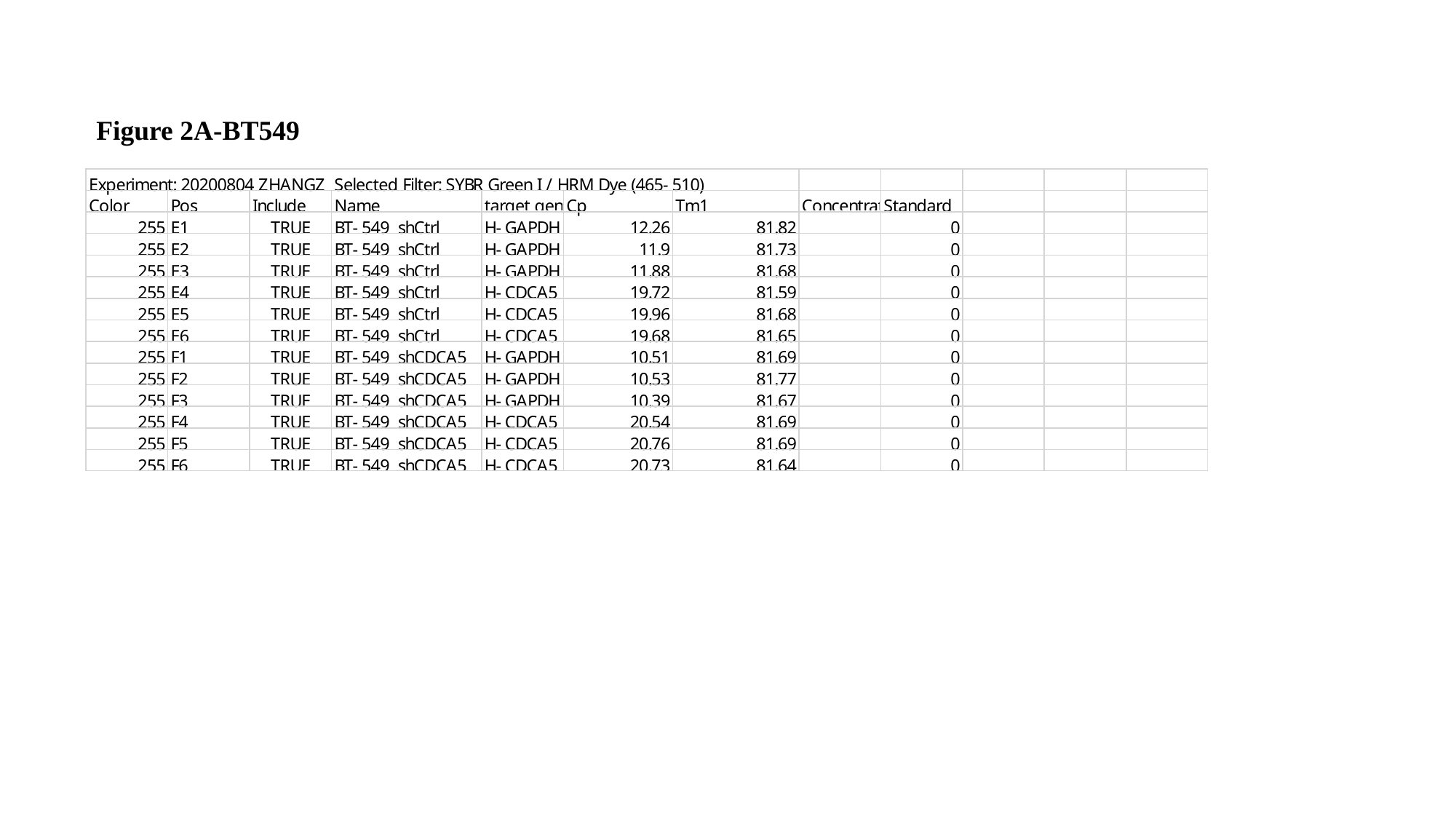

Figure 2A-BT549

## Slide 4
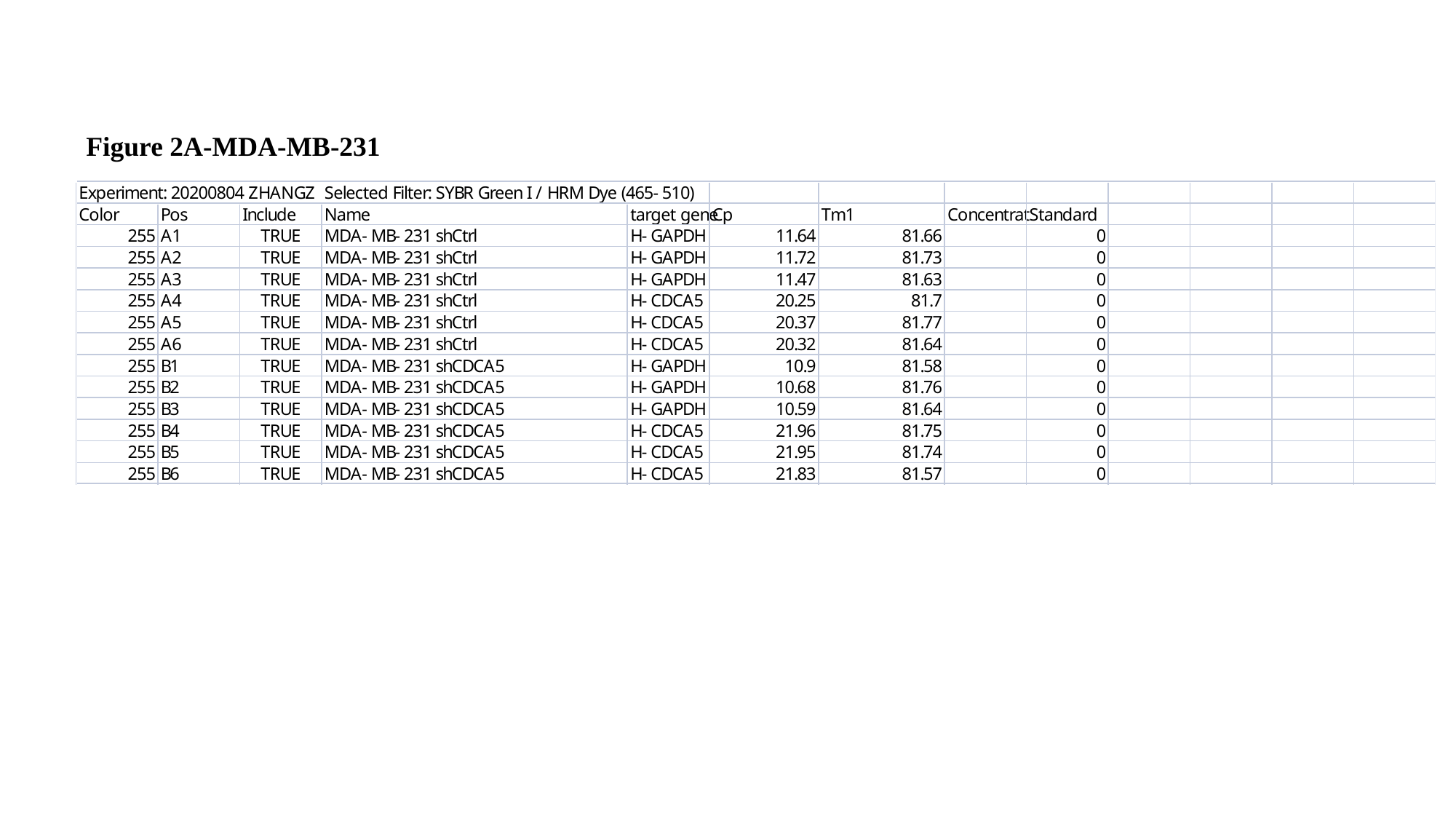

Figure 2A-MDA-MB-231

## Slide 5
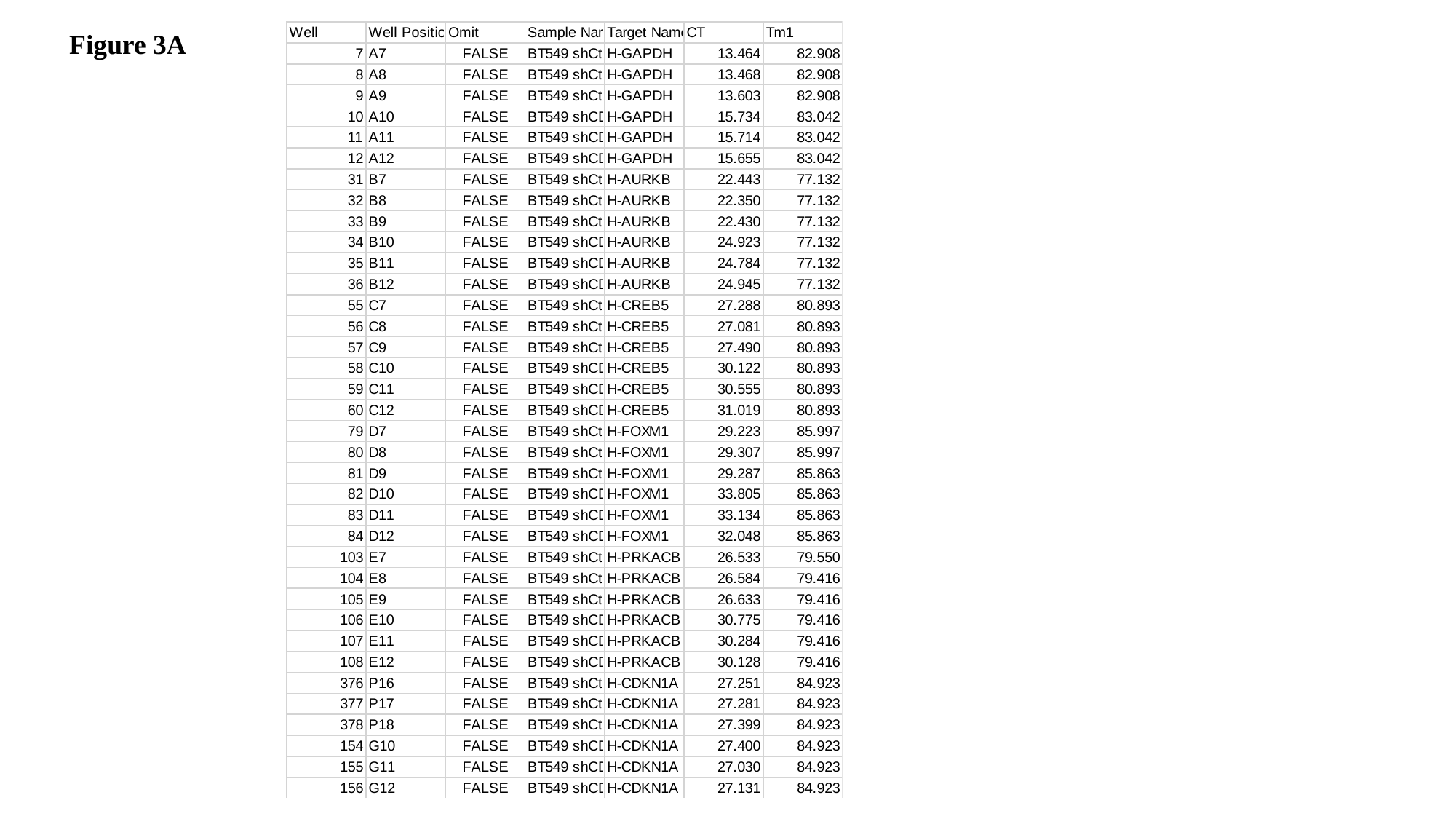

Figure 3A

## Slide 6
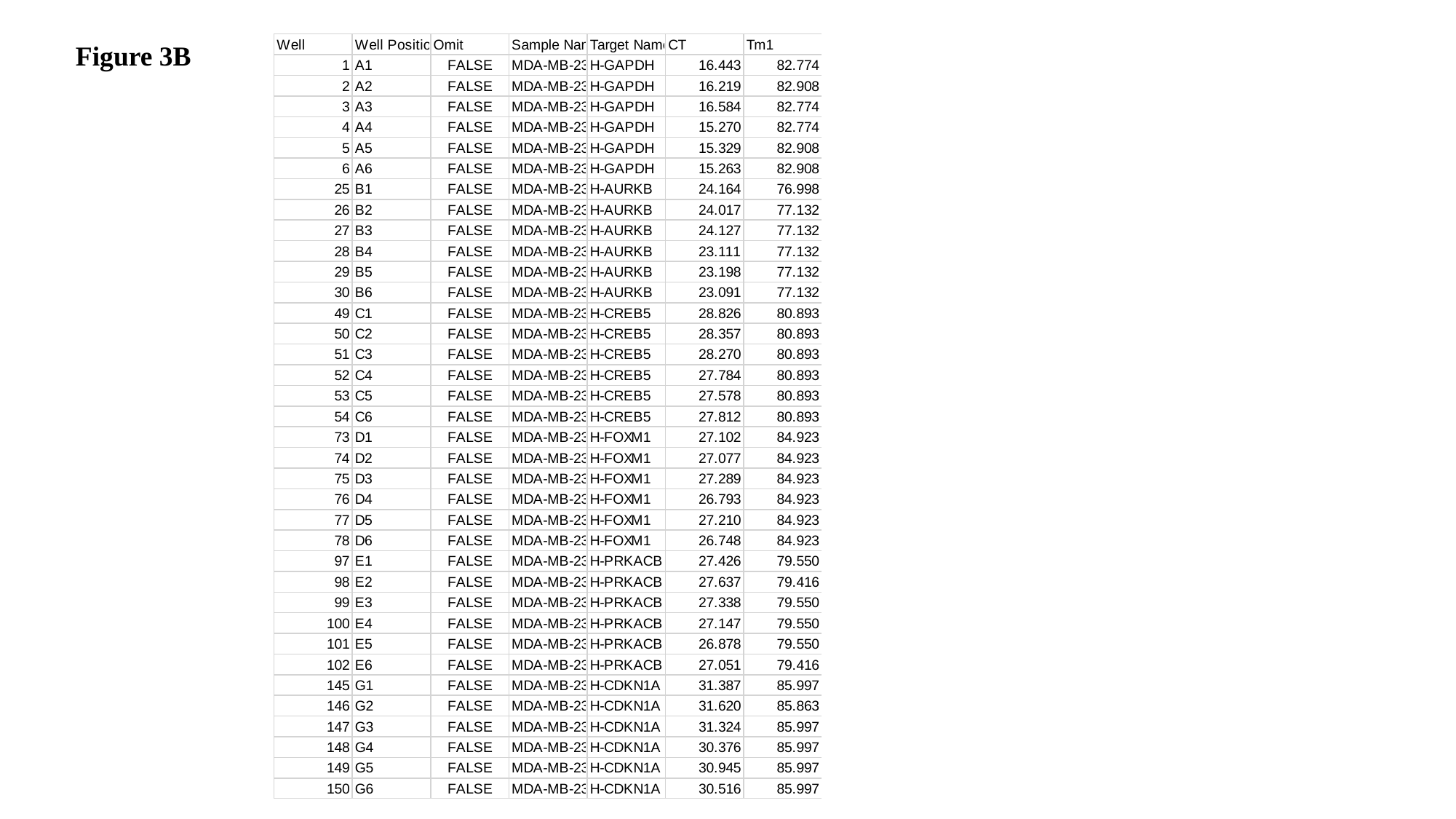

Figure 3B

## Slide 7
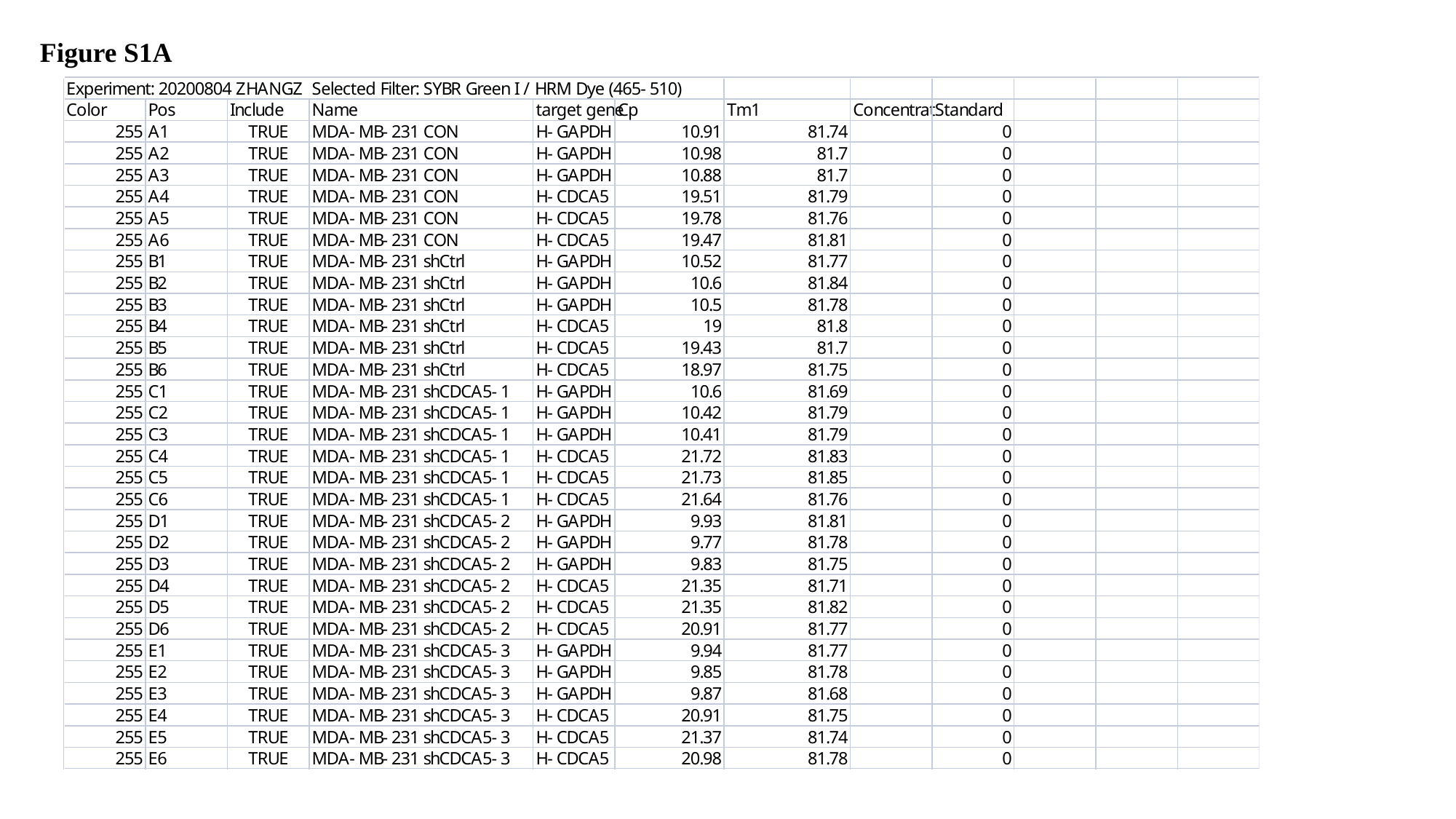

Figure S1A

## Slide 8
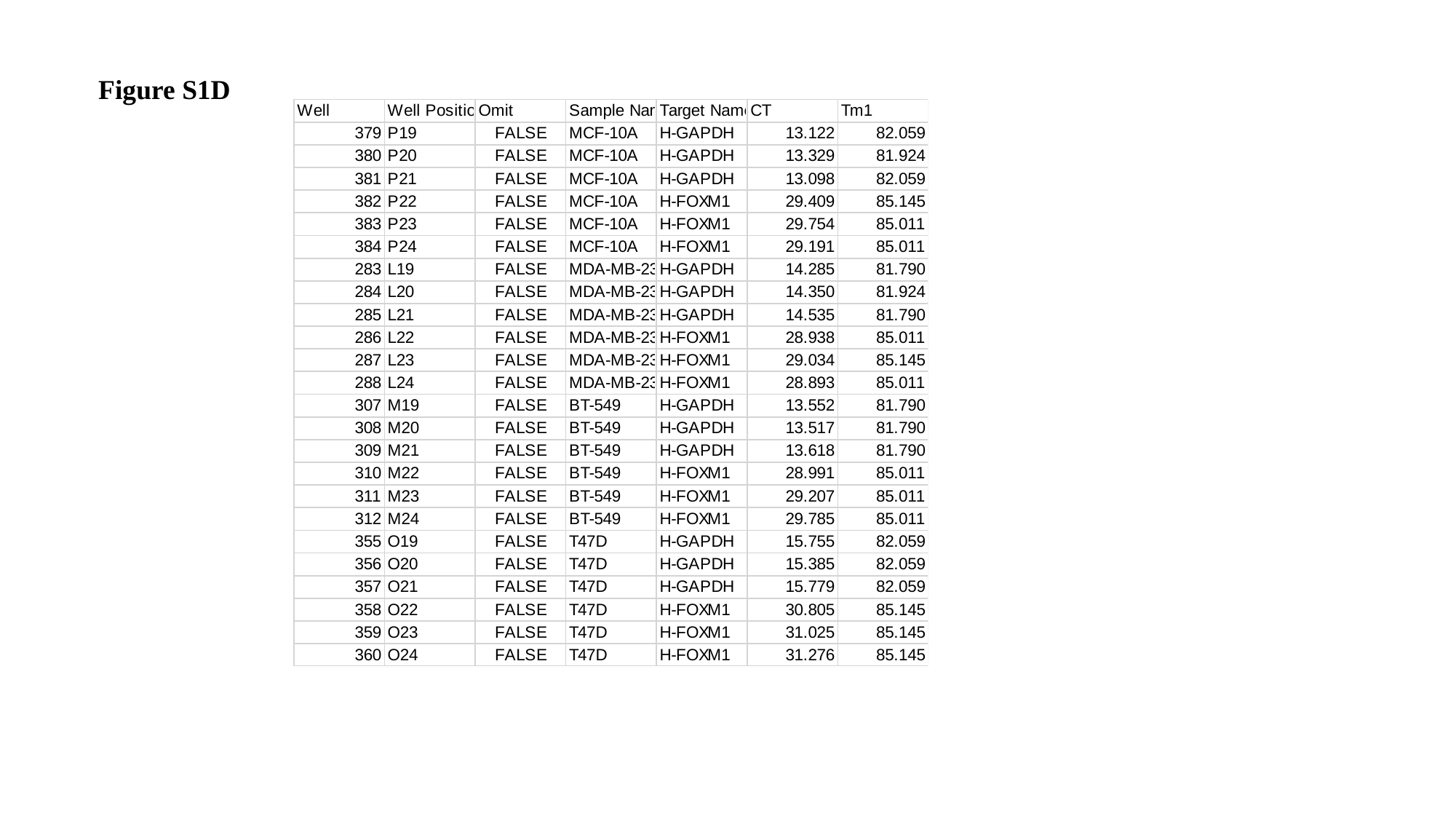

Figure S1D

## Slide 9
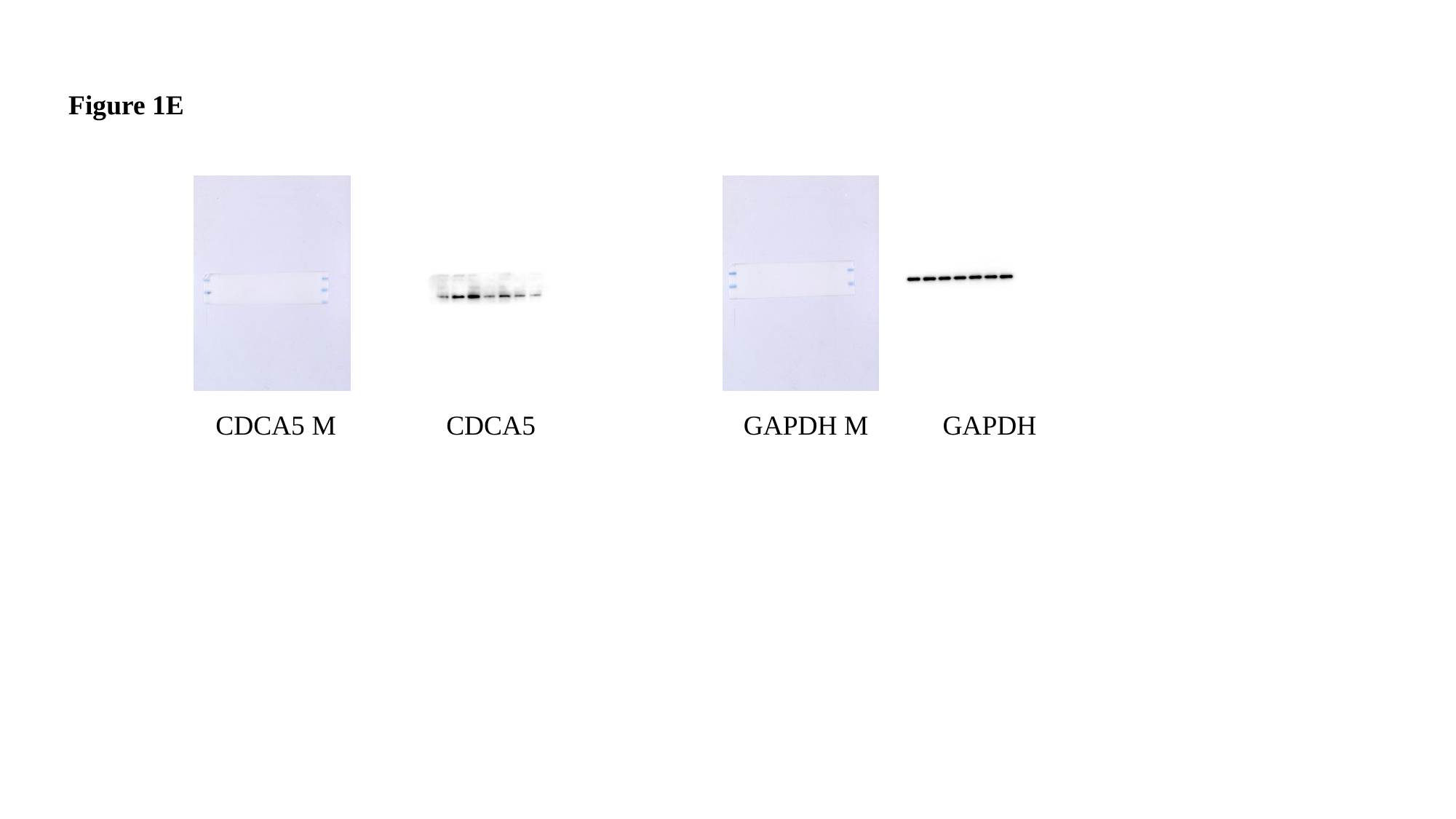

Figure 1E
CDCA5 M
CDCA5
GAPDH M
GAPDH

## Slide 10
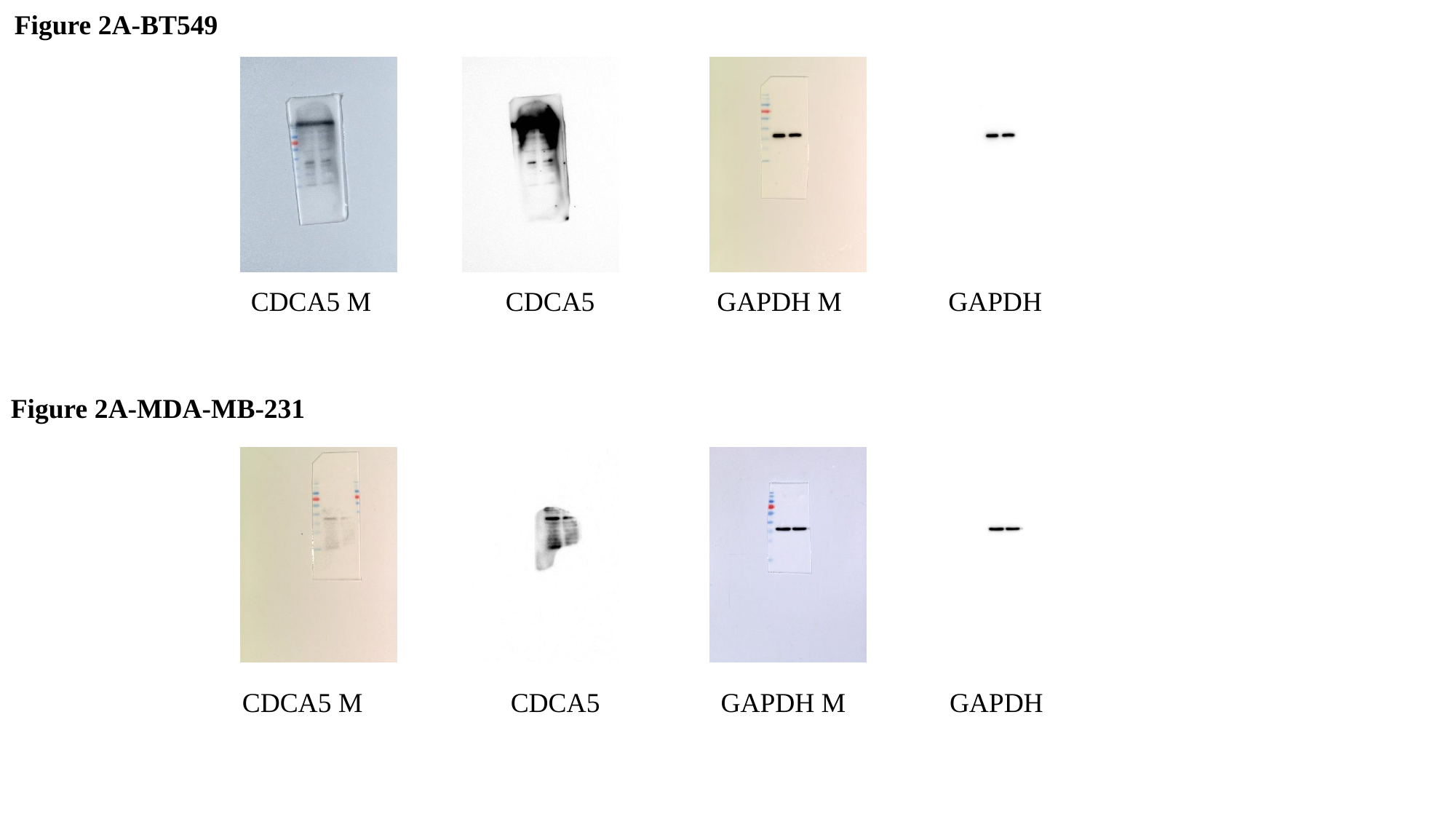

Figure 2A-BT549
CDCA5 M
CDCA5
GAPDH M
GAPDH
Figure 2A-MDA-MB-231
CDCA5 M
CDCA5
GAPDH M
GAPDH

## Slide 11
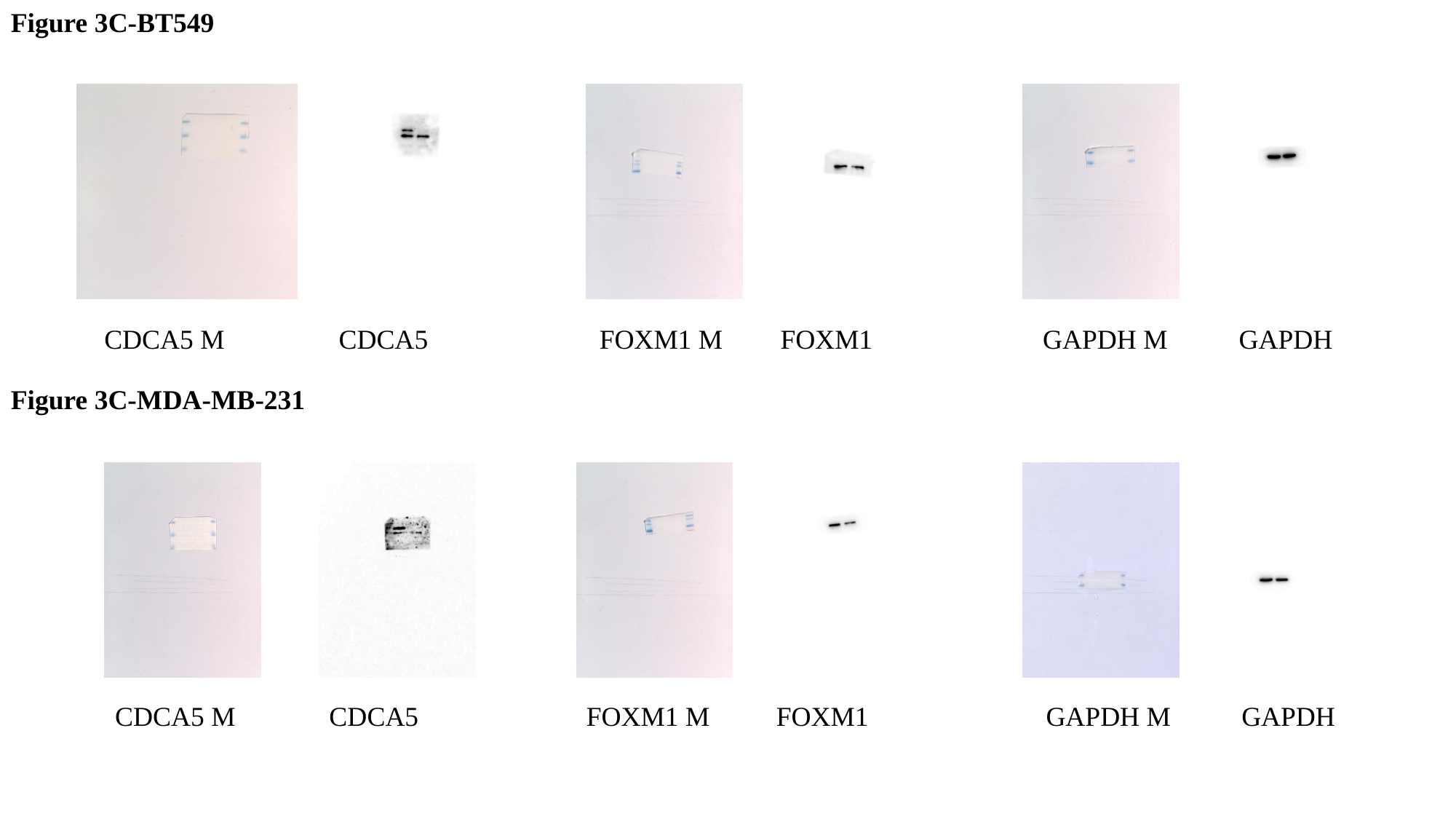

Figure 3C-BT549
CDCA5 M
CDCA5
FOXM1 M
FOXM1
GAPDH M
GAPDH
Figure 3C-MDA-MB-231
CDCA5 M
CDCA5
FOXM1 M
FOXM1
GAPDH M
GAPDH

## Slide 12
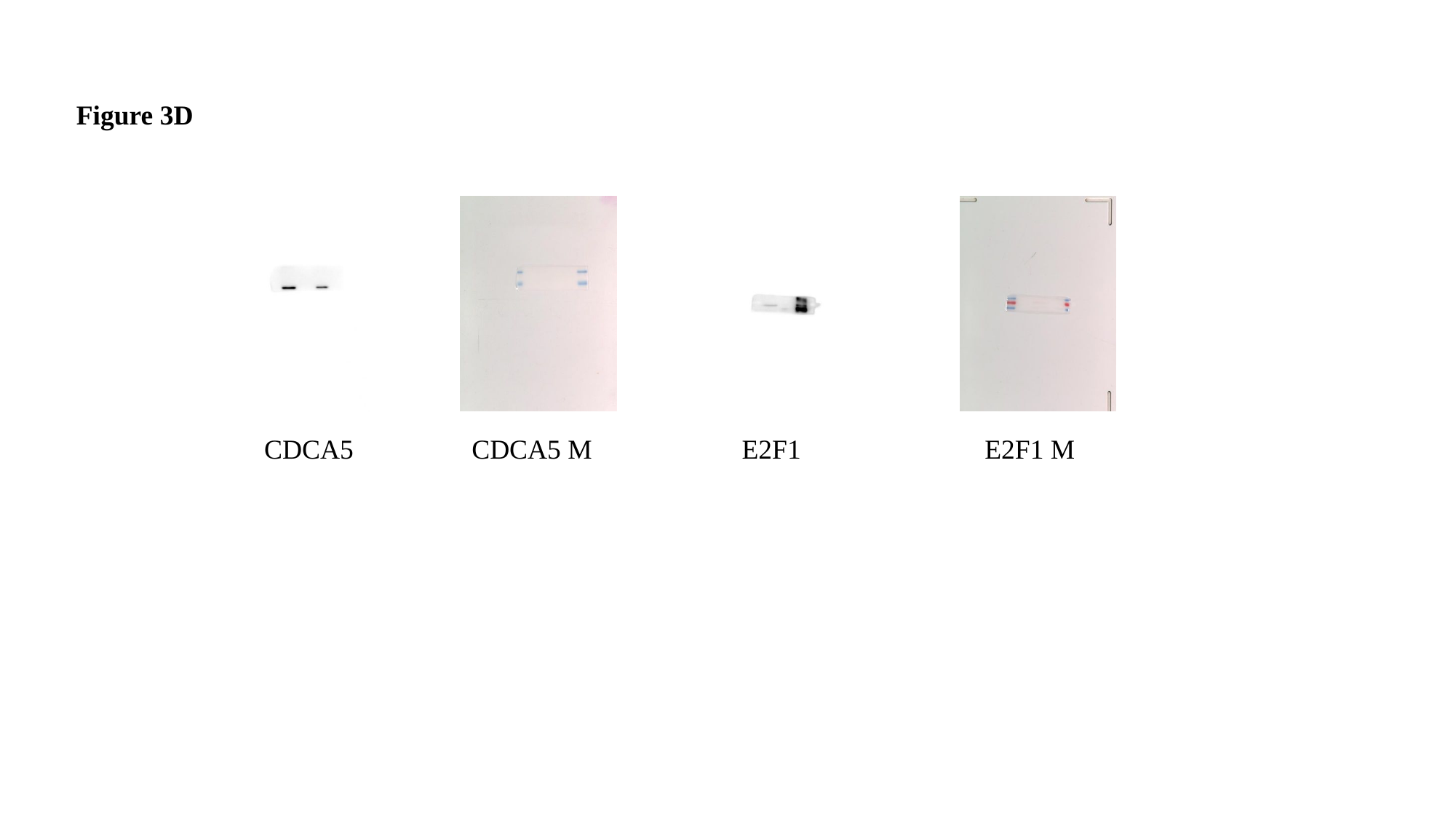

Figure 3D
CDCA5
CDCA5 M
E2F1
E2F1 M

## Slide 13
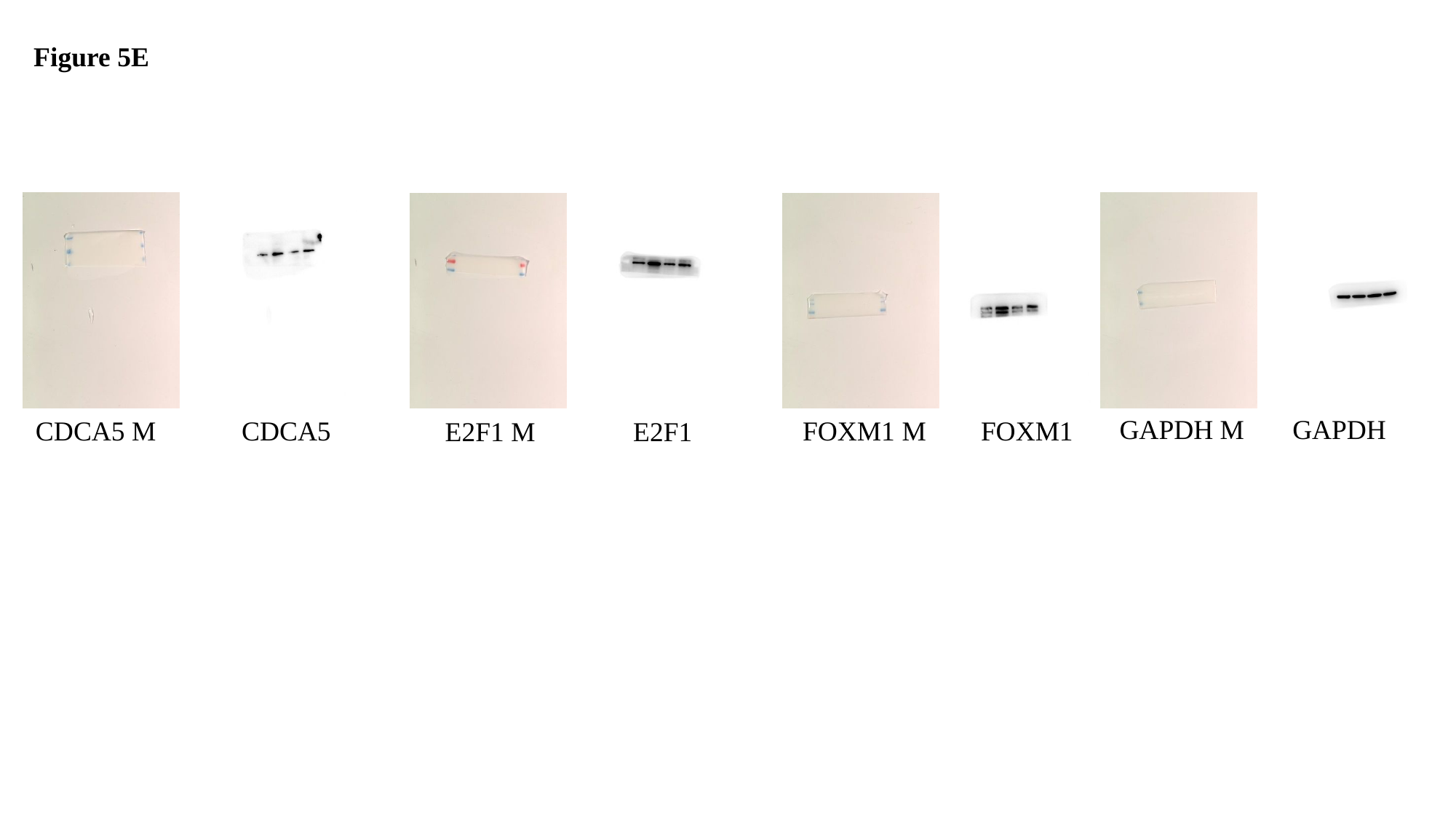

Figure 5E
GAPDH M
GAPDH
CDCA5 M
CDCA5
FOXM1 M
FOXM1
E2F1
E2F1 M

## Slide 14
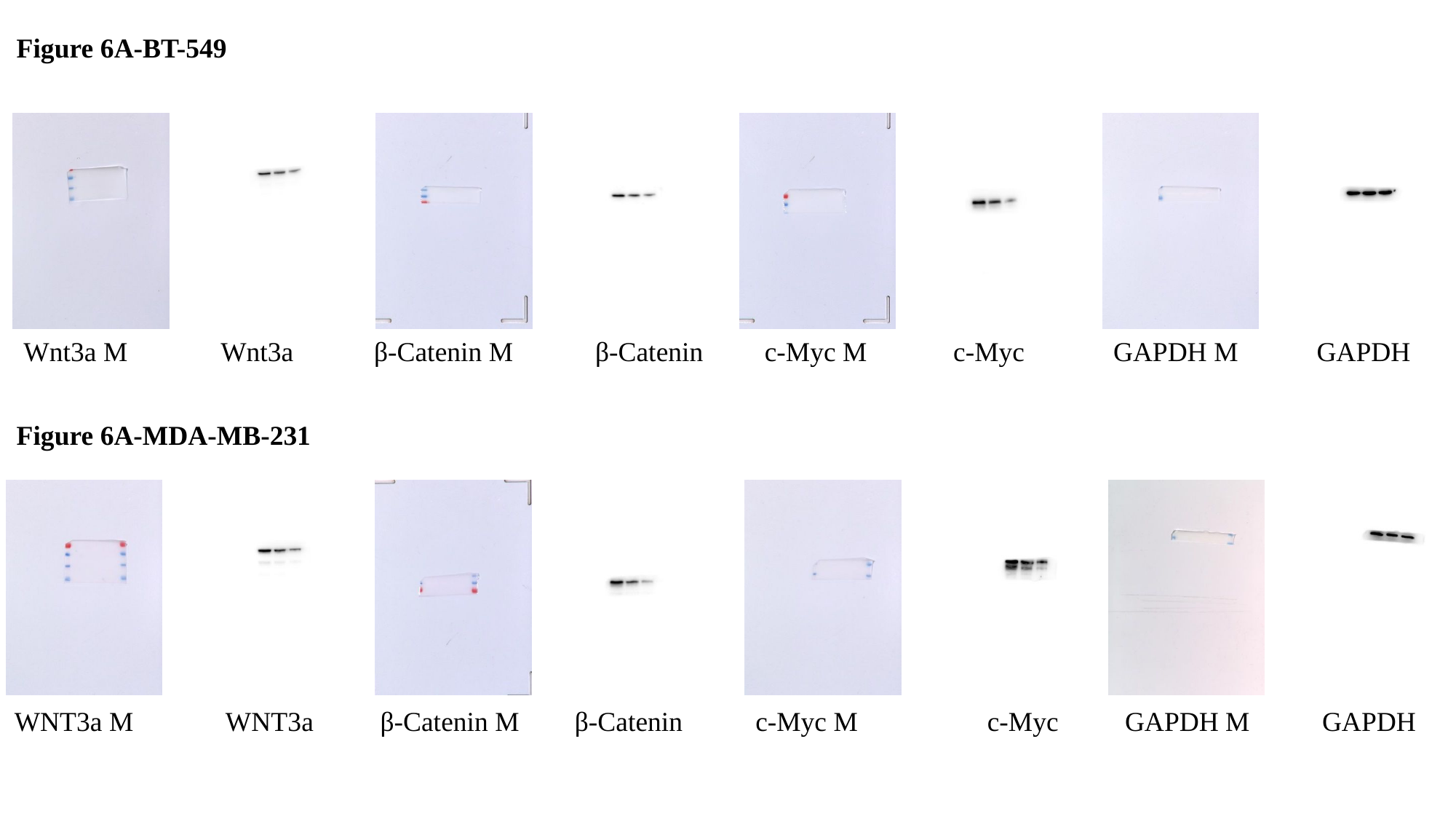

Figure 6A-BT-549
Wnt3a M
Wnt3a
β-Catenin M
β-Catenin
c-Myc M
c-Myc
GAPDH M
GAPDH
Figure 6A-MDA-MB-231
WNT3a M
WNT3a
β-Catenin M
β-Catenin
c-Myc M
c-Myc
GAPDH M
GAPDH

## Slide 15
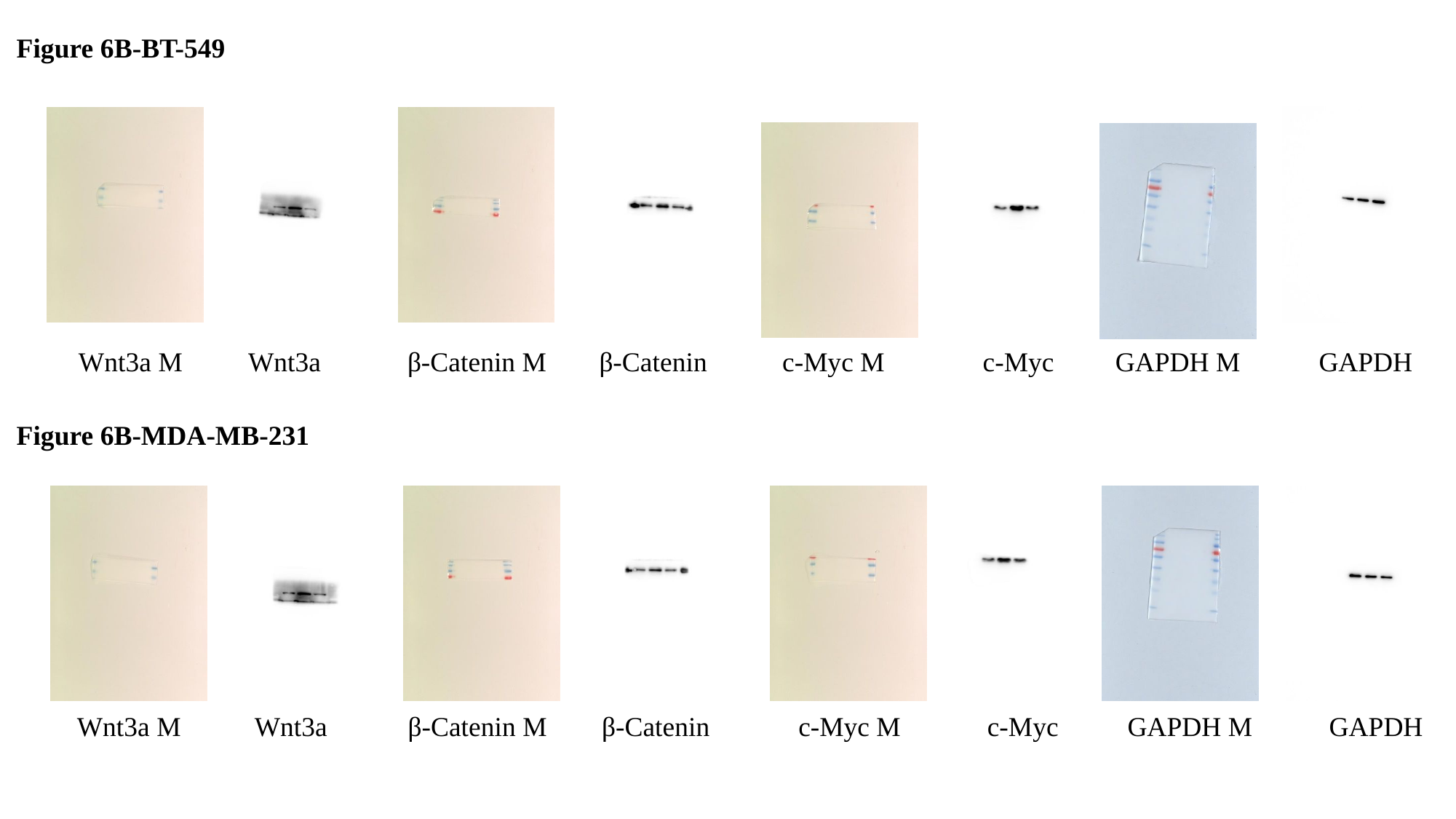

Figure 6B-BT-549
Wnt3a M
Wnt3a
β-Catenin M
β-Catenin
c-Myc M
c-Myc
GAPDH M
GAPDH
Figure 6B-MDA-MB-231
Wnt3a M
Wnt3a
β-Catenin M
β-Catenin
c-Myc M
c-Myc
GAPDH M
GAPDH

## Slide 16
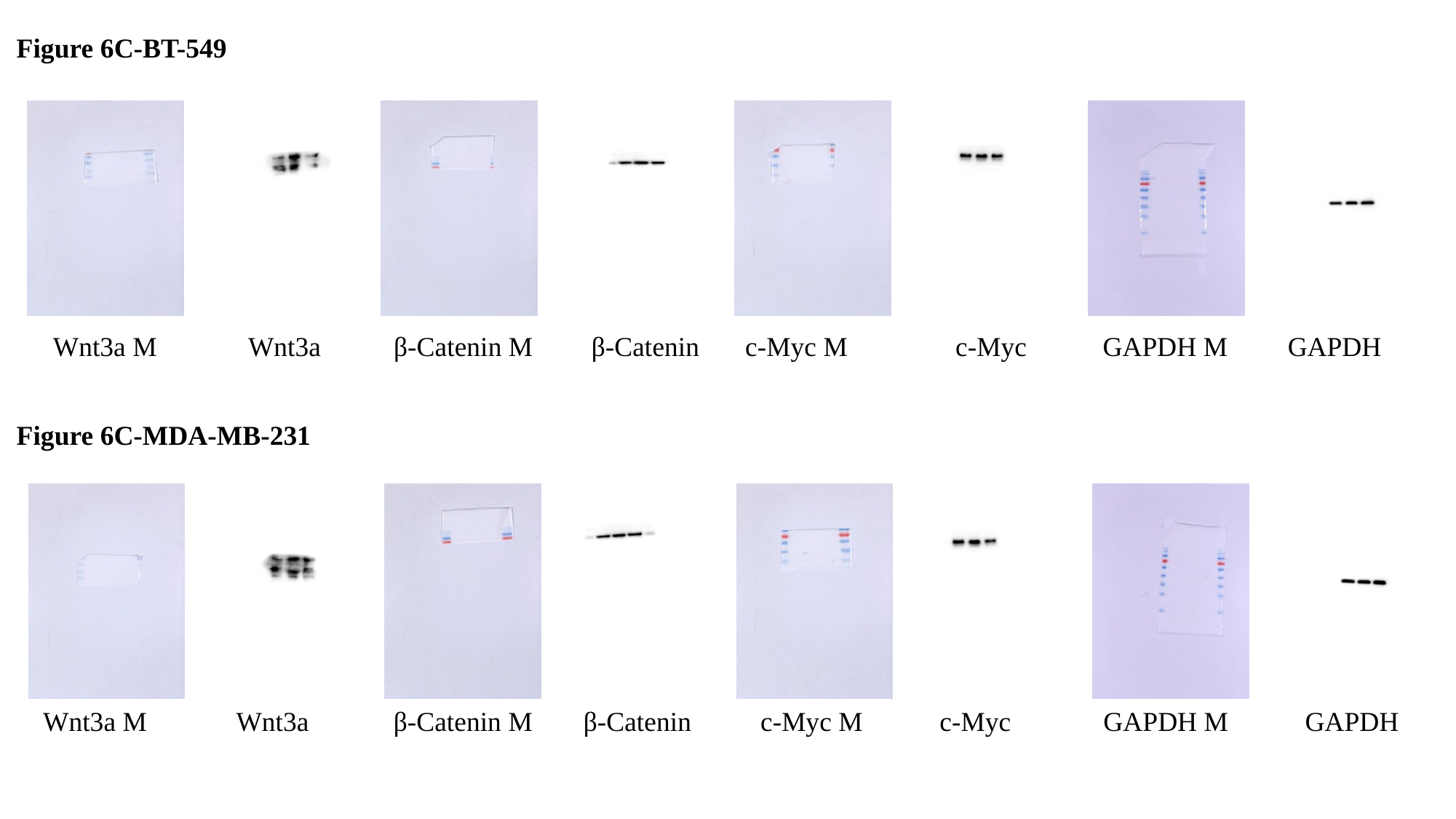

Figure 6C-BT-549
Wnt3a M
Wnt3a
β-Catenin M
β-Catenin
c-Myc M
c-Myc
GAPDH M
GAPDH
Figure 6C-MDA-MB-231
Wnt3a M
Wnt3a
β-Catenin M
β-Catenin
c-Myc M
c-Myc
GAPDH M
GAPDH
